# Still Something to Discover: Novel Insights into *Escherichia coli* Phage Diversity and Taxonomy

**DOI:** 10.3390/v11050454

**Published:** 2019-05-17

**Authors:** Imke H. E. Korf, Jan P. Meier-Kolthoff, Evelien M. Adriaenssens, Andrew M. Kropinski, Manfred Nimtz, Manfred Rohde, Mark J. van Raaij, Johannes Wittmann

**Affiliations:** 1 Leibniz Institute DSMZ–German Collection of Microorganisms and Cell Cultures, 38124 Braunschweig, Germany; ims16@dsmz.de (I.H.E.K.); jan.meier-kolthoff@dsmz.de (J.P.M.-K.); 2 Quadram Institute Bioscience, Norwich Research Park, Colney Lane, Norwich NR4 7UQ, UK; evelien.adriaenssens@quadram.ac.uk; 3 Departments of Food Science and Pathobiology, University of Guelph, Guelph, ON N1G 2W1, Canada; Phage.Canada@gmail.com; 4 Protein Analytics Platform, Helmholtz-Centre for Infection Research (HZI), 38124 Braunschweig, Germany; Manfred.Nimtz@helmholtz-hzi.de; 5 Central Facility for Microscopy, Helmholtz-Centre for Infection Research (HZI), 38124 Braunschweig, Germany; Manfred.Rohde@helmholtz-hzi.de; 6 Department of Macromolecular Structure, Centro Nacional de Biotecnologia CNB-CSIC, 28049 Madrid, Spain; mjvanraaij@cnb.csic.es

**Keywords:** bacteriophage, *Escherichia coli*, taxonomy, diversity, genomics

## Abstract

The aim of this study was to gain further insight into the diversity of *Escherichia coli* phages followed by enhanced work on taxonomic issues in that field. Therefore, we present the genomic characterization and taxonomic classification of 50 bacteriophages against *E. coli* isolated from various sources, such as manure or sewage. All phages were examined for their host range on a set of different *E. coli* strains, originating, e.g., from human diagnostic laboratories or poultry farms. Transmission electron microscopy revealed a diversity of morphotypes (70% Myo-, 22% Sipho-, and 8% Podoviruses), and genome sequencing resulted in genomes sizes from ~44 to ~370 kb. Annotation and comparison with databases showed similarities in particular to T4- and T5-like phages, but also to less-known groups. Though various phages against *E. coli* are already described in literature and databases, we still isolated phages that showed no or only few similarities to other phages, namely phages Goslar, PTXU04, and KWBSE43-6. Genome-based phylogeny and classification of the newly isolated phages using VICTOR resulted in the proposal of new genera and led to an enhanced taxonomic classification of *E. coli* phages.

## 1. Introduction

Though being a ubiquitous and most of the time harmless commensal, *Escherichia coli* (*E. coli*) is among the most common pathogens known to both human and veterinary medicine. Moreover, *Shigella* spp., the causative agent of human shigellosis, is directly imbedded within *E. coli* diversity [1]. Many strains cause various diseases in humans and animals due to different mechanisms of pathogenicity. According to the site of infection, those diseases can be grouped as intestinal, e.g., causing diarrhea, or extraintestinal, e.g., causing urinary tract infections (UTI) or septicaemia [2]. The intestinal group can be further differentiated into six different pathotypes [3,4], namely enteropathogenic *E. coli* (EPEC), enterohaemorrhagic *E. coli* (EHEC), enterotoxigenic *E. coli* (ETEC), enteroinvasive *E. coli* (EIEC), enteroaggregative *E. coli* (EAEC), and diffusely adherent *E. coli* (DAEC) dependent on phylogeny or presence of virulence factors. In case of the extraintestinal *E. coli,* they are classified based on the diseases that are associated with them, e.g., uropathogenic *E. coli* (UPEC) or neonatal meningitis-associated *E. coli* (NMEC) [5]. A genome-based analysis of a comprehensive *E. coli* dataset proposed a subdivision of *E. coli* into five subspecies and 14 phylogroups [1].

In poultry, *E. coli* is a major causative agent of colibacillosis, which creates significant economic losses and is commonly treated by antibiotics, which in turn promotes the selection of multidrug-resistant (MDR) bacteria. Since antibiotic resistance is also a major concern in *E. coli*, extended-spectrum beta-lactamase ESBL-producing *Enterobacteriaceae* are also on the World Health Organization (WHO) priority list for pathogens against which solutions must urgently be developed. Thus, bacteriophages have resurfaced as one putative alternative to antibiotics [6] and some reports of phage therapy to treat *E. coli* infections have already been reported for veterinary and human medicine [7,8].

Bacteriophages are assumed to be the most abundant biological entities on earth with an estimated number of 4.8 × 10^31^ phage particles in the whole biosphere [9]. This abundance makes them global players in different ecological processes, such as biogeochemical cycling [10,11,12]. Besides their impact on ecology and evolution, they also played an important role in the development of modern molecular biology. Many pioneering experiments based on bacteriophages and their interaction with their hosts were performed that resulted in the discovery of basic aspects of today’s molecular biology, such as the proof of DNA as the hereditary material (Hershey and Chase, 1952), restriction and modification of DNA (Arber, 1960s), or phage Lambda as a model for gene regulation [13]. Those well-studied workhorses, such as λ, members of the T-series, P1, or N4-like phages, are still strongly used in research for different questions.

However, although the number of sequenced phage genomes is constantly rising [14], only a relatively small number is well-characterized and taxonomically classified [15,16]. The International Committee on Taxonomy of Viruses (ICTV) currently classifies caudoviral *Escherichia* phages into 37 genera and 157 species. N.B. ICTV does not classify viral strains, i.e., those phages which show ≥95% DNA sequence identity [17,18]. Furthermore, though a lot of new relatives of previously classified phages have been isolated over the years, there are still genomic orphans in the databases. In regard to *Escherichia* phages, several studies have recently been performed looking at the diversity of those phages in different habitats, such as water or even food [19,20]. This study provides further insight into the diversity of *E. coli* phages via morphology and genome-based phylogeny and classification. Therefore, samples from different sources, such as surface water, manure, sewage, or animal feces, were used to isolate phages on a diverse set of *E. coli* strains with different serotypes from mostly human or animal origin.

## 2. Materials and Methods

### 2.1. Bacterial Strains and Growth Conditions

*E. coli* isolates DSM 101101–101142 come from clinical material around Hameln (Germany), DSM 103242–103251 originate from poultry carcass skin from France, Belgium, Netherlands, and Germany, and APEC (avian pathogenic *E. coli*) strains (DSM 103254–103266) were isolated in Germany. Together with the laboratory strain DSM 498, they were used for phage isolation, propagation, and host range analysis. Serotypes of the isolates were determined according to the Ørskov typing scheme [21] and the information can be found in Table S1. To ensure the diversity of the strains used for host range analysis, a selected number of strains was characterized by Alere Technologies GmbH *E. coli* PanType [21,22]. The *E. coli* isolates were grown in lysogeny broth (LB) (2.5% Miller’s LB Broth Base^TM^ powder (Invitrogen, Carlsbad, CA, USA) or on agar plates of LB medium supplemented with 1.5% bacteriological agar No. 1 (*w*/*v*) (Oxoid^TM^, Thermo Fisher Scientific, Waltham, MA, USA)) overnight at 37 °C unless stated otherwise. To test the specificity of the phages KWBSE43-6 and Goslar, we used different *Klebsiella* strains from the collection of the German Collection of Microorganisms and Cell Cultures (DSMZ) for an extended host range analysis. The growth conditions are summarized in Table S2.

### 2.2. Phage Isolation, Purification, and Propagation

Phages were isolated from different samples using 29 different *E. coli* strains between November 2015 and August 2016 (Table S3).

After centrifugation, samples were filtrated (membrane syringe filter 0.45 µm, Sartorius (Germany)) and mixed with equal amounts of double-concentrated LB broth and 1/20 volume logarithmic growing host strain. After incubation at 37 °C overnight, cells were sedimented by centrifugation and the supernatant was filtrated. To isolate phages, appropriate dilutions of the enrichment were mixed with soft-agar (2.5% Miller’s LB Broth Base™ powder (Invitrogen, Thermo Fisher Scientific, USA) and 0.3–0.7% bacteriological agar No. 1 (*w*/*v*) (Oxoid^TM^, Thermo Fisher Scientific, USA)) containing the corresponding host and overlaid on an agar plate. After incubation at 37 °C for 12–18 h, single plaques were suspended in SM buffer (100 mM NaCl, 8 mM MgSO_4_, 50 mM Tris-HCl, pH 7.5 (Merck, Germany)) using a pipette tip followed by striking on a double agar plate. At least four consecutive single plaque isolates were processed for a pure phage preparation, which was used for further propagation. In general, logarithmic growing cultures were infected with phages at multiplicicty of infection MOI 0.1, and incubated for 10 min at room temperature and then at 37 °C and 123 rpm until the lysis was completed or for 5 h. After centrifugation and filtration, the titer of the lysate was determined using the double agar method and the lysate was stored at 10 °C.

### 2.3. Purification via CsCl Gradient and Morphological Analysis via Transmission Electron Microscopy

For preparation of the CsCl gradient, 300 mL high titer lysates of our phages (>10^9^ plaque forming units (PFU)/mL) were centrifuged for 2 h at 16,000 rpm (Sorvall RC6Plus, F21S 8 × 50Y) in order to concentrate the phages. Phages were resuspended in 2 mL SM buffer buffer (100 mM NaCl, 8 mM MgSO4, and 50 mM Tris-HCL (pH 7.5)). After incubation at room temperature for 2 h, the phages were purified by ultracentrifugation in a discontinuous CsCl gradient (0.5 mL CsCl solution with densities of 1.6, 1.5, 1.4, and 1.3) for 2 h at 15 °C and 35,000 rpm in an SW 60 Ti rotor. Phage bands were then removed and dialyzed against SM buffer again [23].

For TEM analysis, phages were prepared for analysis as previously described [24]. Briefly, phages were allowed to adsorb onto thin carbon support films. Afterwards, they were negatively stained with 2% (*w*/*v*) aqueous uranyl acetate, pH 5.0. Samples were examined in a Zeiss EM 910 or Zeiss Libra120 Plus transmission electron microscope (Carl Zeiss, Oberkochen, Germany) at an acceleration voltage of 80 kV/120 kV at calibrated magnifications using a crossed line grating replica. Size determination of heads and tails was performed using ITEM Software (Olympus Soft Imaging Solutions, Münster) from at least 3–10 different phage particles and further classification was done according to Ackermann [25]. Based on their morphotypes, phages were then named according to Adriaennsens and Brister [17], e.g., *Escherichia* phage R5505, which revealed characteristics of a myovirus that was named vB_EcoM_R5505. For convenience, only the short names of the phages are used in the text.

### 2.4. Host Spectrum

The phage host range was determined by spotting serial dilutions on double agar plates containing 100 µL of a logarithmic culture of the potential host. After incubation for 14–16 h at 37 °C, the plates were examined for lysis. The host was classified as sensitive when single plaques could be detected.

### 2.5. DNA Isolation and Sequencing

DNA from phage lysates was extracted by phenol-chloroform and precipitated by 3 M sodium acetate and 100% ethanol as previously described [21] or following the manufacturer’s instructions of the Phage DNA Isolation Kit (Norgen Biotek). Briefly, 300 mL high titer lysate (10^10^ PFU/mL) was precipitated with 10% polyethylene glycol 8000 (Merck, Germany) and 1 M sodium chloride (Merck) followed by centrifugation for 30 min at 10,000 rpm (Sorvall RC 6 Plus with rotor F21S 8 × 50 y, Thermo Scientific^TM^, USA). The pellet was resuspended in 2–4 mL SM-buffer and treated with 10-fold reaction buffer (100 mM Tris-HCl (pH 7.5), 25 mM MgCl_2_, und 1 mM CaCl_2_, Thermo Fisher Scientific, USA), 0.2 mg/mL RNase A (Thermo Fisher Scientific, USA), and 0.002 U/µL DNase I (Thermo Fisher Scientific, USA) followed by incubation overnight at 37 °C and 300 rpm in a Thermomix (Eppendorf, Germany). DNA was isolated using phenol-chloroform extraction and precipitated by 3 M sodium acetate and 100% ethanol. After incubation for 15 min at −80 °C, DNA was pelleted by centrifugation and washed twice with 70% ethanol. The pellet was air-dried and solved in 50–200 µL TE-buffer (10 mM Tris-HCl, 1 mM EDTA, pH 8, Merck, Germany). The concentration was determined using the Qubit^®^ dsDNA HS Assay Kit (Thermo Fisher Scientific, USA) following the manufacturer’s instructions.

### 2.6. Library Preparation and Whole Genome Sequencing

Phage genomes were sequenced using PacBio RSII and Illumina MiSeq technologies. A SMRTbell^TM^ template library was prepared according to the instructions from Pacific Biosciences following the Procedure & Checklist Greater than 10 kb Template Preparation and Sequencing. Briefly, for preparation of 10 kb libraries, ~4 µg of each phage DNA was sheared using a Covaris S220 focused-ultrasonicator (Woburn, MA, USA) according to the manufacturer´s instructions. DNA was end-repaired and ligated overnight to barcoded SMRTbell^TM^ adapters applying components from the DNA/Polymerase Binding Kit P6 from Pacific Biosciences. Reactions were carried out according to the manufacturer´s instructions. Seven to eight SMRTbell^TM^ templates were combined in equimolar part. Each sample was either Exonuclease treated for removal of incompletely formed reaction products or pulsed-field gel electrophoresis was carried out using a BluePippin^TM^ to select for fragments greater than 4 kb. The latter was performed according to the manufacturer´s instructions (Sage Science). Conditions for annealing of sequencing primers and binding of polymerase to a purified SMRTbell^TM^ template were assessed with the Calculator in RS Remote, Pacific Biosciences. Single molecule real time (SMRT) sequencing was carried out on the PacBio RSII (Pacific Biosciences) taking one 240-min movie for each SMRT cell. In total, one SMRT cell was run for each assay. Alternatively, libraries were prepared as described by Baym et al. [26]. An amount of 0.5 ng DNA was enzymatically fragmented using the Nextera DNA Library Preparation Kit (Illumina, FC-121-1030) for 10 min at 55 °C. After addition of 11 µL KAPA-master mix (Library Amplification Kit, Kapa Biosystems) and 4.4 µL of each index primer, the reaction mixture was incubated according the following program: 3 min at 72 °C, 5 min at 98 °C, 13× (10 s at 98 °C, 30 s at 62 °C, 30 s at 72 °C), 5 min at 72 °C. For PCR clean-up and size selection, the PCR product was incubated with magnetic beads (Ampure XP, Beckman Coulter). The pool of DNA libraries was adjusted to 4 nM and sequenced using MiSeq technology (Reagent Kit v3, 600-cycle, MS-102-3003, Illumina).

### 2.7. SDS-PAGE Analysis of Phage Proteins

Phages from CsCl-gradient-purified lysates were heated for 7 min at 95 °C with loading buffer (0.1 M DTT, 0.01% bromophenol blue sodium salt, 1.3% SDS, 22% glycerol, 0.13 M TRIS) at the ratio 4:1. Proteins were separated by electrophoresis on 15% SDS-PAGE gels (running gel: 15% acrylamide-,*N*,*N*′-methylenebisacrylamide-solution ratio 37.5:1 (Carl Roth, Germany), 0.1% SDS, 0.1% APS in 0.375 M TRIS-HCl, pH 8.8; stacking gel: 10% acrylamide-,*N*,*N*′-methylenebisacrylamide-solution ratio 37.5:1, 0.1% SDS, 0.1% APS in 0.05 M TRIS-HCl, pH 6.8) in SDS-PAGE buffer (0.2 M glycine, 0.025 M TRIS, 1% SDS, pH 8.3). Gels were incubated in fixation solution (50% methanol and 10% acetic acid) for 30 min, protein bands visualized by staining with 0.25% Coomassie R250 in fixation solution for 1 h, and background color was removed with decolorizer solution (5% methanol, 7.5% acetic acid) overnight. Prominent protein bands were cut in slices, which were washed in distillated water overnight and afterwards dried in air.

### 2.8. Time-Limited Digestion with BAL 31

For further analysis and to obtain a hint at a putative circular permutation of the genome, phage DNA was treated with exonuclease BAL 31 and restriction enzymes as previously described [27]. 

### 2.9. MALDI-TOF Analysis

Peptide fingerprinting analysis by MALDI-TOF analysis was performed as described previously [28]. Briefly, excision of stained protein bands was followed by two washing steps with water for 5 min. After that, gel slices were dehydrated with 200 μL of 50% acetonitrile for 5 min, then reduced using 20 mM DTT in 200 μL of 0.1 M NH_4_HCO_3_ for 30 min at 56 °C and dehydrated again followed by rehydration with 55 mM iodoacetamide in 0.1 M NH_4_HCO_3_ and incubated for 30 min at room temperature (RT) in the dark. Dehydrated gel pieces were completely dried in a SpeedVac concentrator for 10 min. They were reswelled in 50 μL of digestion solution containing trypsin, followed by incubation overnight at 37 °C. Peptides were extracted by adding 50 μL of acetonitrile, followed by incubation with vigorous shaking for 15 min at 37 °C. After discarding the supernatants, gel slices were treated with 50 μL of 5% formic acid with vigorous shaking for 15 min at 37 °C. Afterwards, 50 μL of acetonitrile was added, followed again by incubation with vigorous shaking for 15 min at 37 °C. Supernatants were combined with the removed supernatants and concentrated using a SpeedVac concentrator. Forty microliters (40 μL) of 32% methanol/0.25% HCOOH was added, followed by ultrasound treatment for 3 min and analysis by MALDI Ultraflex-TOF/TOF. Results were compared with a database of predicted phage proteins using Mascot (an in-house system).

### 2.10. Genome Sequencing, Assembly, and Annotation

For PacBio RS II sequencing, phage genome sequence assembly was performed using the “RS_HGAP_Assembly.3” protocol included in SMRTPortal version 2.2.0., applying standard parameters as previously described [24]. Sequences generated with Illumina technology were assembled using SPAdes (3.12.0) with the careful option (55kmer) [29]. For all phages, a single contig was obtained that was linearized due to recognition of distinct start and end points in the phage assemblies where the majority of sequences ended abruptly. If ends could not be detected, such as for T4-like phages with circularly permuted genomes, assembled sequences were arranged according to the genomic organization of closely related and already published phages. A quality check of the final phage genomes regarding overall coverage was done using SMRT View and IGV [30]. All phage genomes were annotated with Prokka 1.8 using different databases (Markov model profile databases, including Pfam and TIGRFAMs). A search was performed using hmmscan from the HMMER 3.1 package [31]). Additionally, the pVOG database was included our analysis [32]. Genomes were then manually curated with Artemis [33]. Intergenic genome regions were analyzed for putative transcriptional regulation elements with ARNOLD [34]. A search for tRNA genes was done with the tRNAscan-SE program v1.2.1 [35]. Homology assignments were based on amino acid sequence alignment searches (BLASTP against non-redundant protein sequences and the UniProtKB/Swiss-Prot database) and were accepted only if the statistical significance of the sequence similarities (E value) was less than 1 × 10^−5^, the percentage query cover was ≥60%, and the percentage identity between the aligned sequences was ≥35%.

### 2.11. VICTOR Analysis

The complete dataset of newly isolated phages, including reference phage species, was subjected to a state-of-the-art genome-based analysis using VICTOR [36], the Virus Classification and Tree Building Online Resource (freely available at https://victor.dsmz.de). VICTOR was shown to yield either nucleotide- or amino-acid-based phylogenies having a high agreement with the ICTV classification and the majority of taxa was well-supported as monophyletic. Recent applications, for example, include the successful classification of newly isolated roseophages into a novel genus and subfamily [37]. VICTOR makes extensive use of the Genome-BLAST Distance Phylogeny (GBDP) method [38] under settings recommended for prokaryotic viruses [36]. The phylogenetic trees (with branch support) and taxon boundaries at the species, genus, subfamily, and family level, as reported and recommended by VICTOR, were finally visualized with iTOL [39] after preparing the annotation with the R program table2itol (https://github.com/mgoeker/table2itol). For analysis with VICTOR, BLASTn analysis of our phage genomes against the database of tailed phages (taxid:28883) was performed first to identify related phages with a high identity score (October 2018). When phages belonged to a yet unclassified group, we tried to include all of them in further analyses. For already classified genera with a high number of members, such as *Tequatrovirus* or *Tequintavirus*, we chose the type phages and best hits to maintain dataset sizes still suitable for visualization. Before the VICTOR analyses, reference phages HX01 and PDX were reannotated using Prokka to provide comparable numbers of coding sequences (CDS) since the submitted GenBank file (MG963916, JX536493) apparently missed several CDS.

## 3. Results and Discussion

### 3.1. Morphological Analysis via TEM

**Figure 1:**
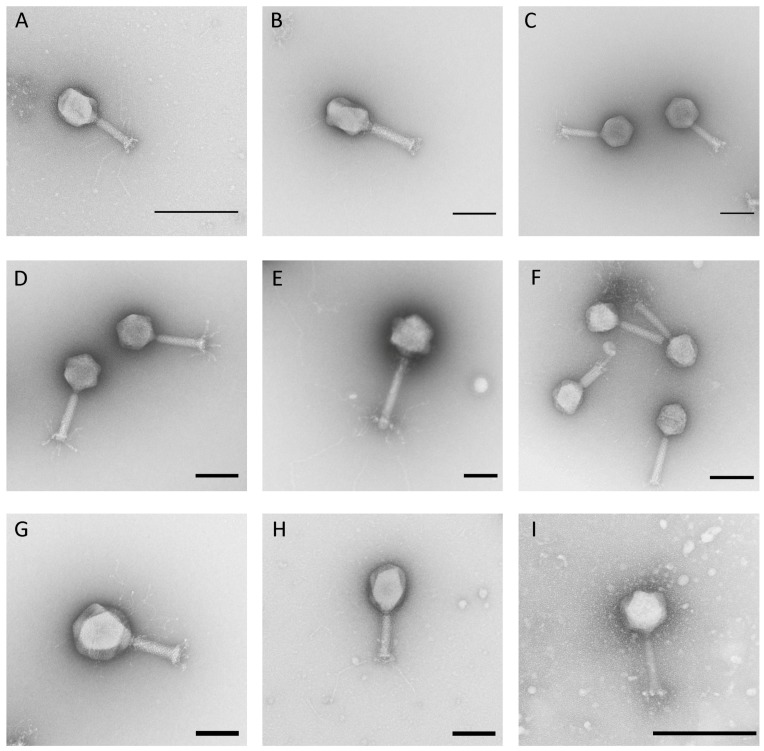
Electron micrographs of isolated phages G53 (**A**), G50 (**B**), Schickermooser (**C**), HdK5 (**D**), Goslar (**E**), WFH (**F**), G17 (**G**), G4507 (**H**), and KWBSE43-6 (**I**) of the myoviruses. Negative staining (2% (*w*/*v*) uranyl acetate, pH 5.0). Bars represent 100 nm, except for A and I (200 nm).

**Figure 2:**
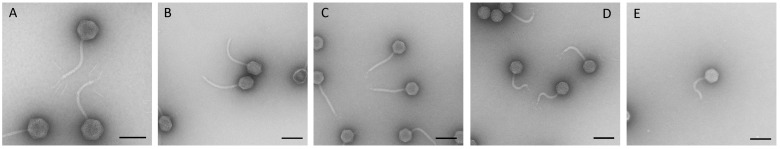
Electron micrographs of isolated phages HASG4 (**A**), HdK1 (**B**), MM01 (**C**), PTXU06 (**D**), and G29-2 (**E**) of the siphoviruses family. Negative staining (2% (*w*/*v*) uranyl acetate, pH 5.0). Bars represent 100 nm.

**Figure 3:**
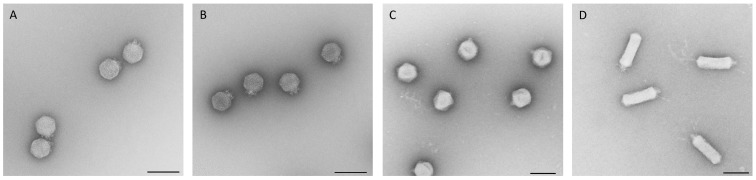
Electron micrographs of isolated phages KAW1A4500 (**A**), R4596 (**B**), PTXU04 (**C**), and WFI101126 (**D**) of the podoviruses. Negative staining (2% (*w*/*v*) uranyl acetate, pH 5.0). Bars represent 100 nm.

All newly isolated phages of this study were morphologically analyzed via transmission electron microscopy. This analysis revealed that all belong to the order *Caudovirales* with representatives of three different morphotypes. The majority belonged to the myoviruses (70%) and siphoviruses (22%). Only four isolates (8%) showed a characteristic morphology of a podovirus, indicating that a substantial diversity of phages was isolated during this study. Among the isolated myoviruses, different tail lengths, varying between ~100 nm and ~220 nm, and various tail fibers, their head widths ranging from 70 to ~130 nm (Figure 1, Table 1), were detected. Analysis of the isolated siphoviruses revealed isometric head widths ranging from ~60 to ~80 nm and tail lengths ranging from 140 to ~220 nm. Some phages, such as, for instance, phage HDK1, revealed slightly prolate heads. Most of the examined siphoviruses displayed relatively flexible tails and also tail fibers at the end (Figure 2). Finally, phages KAW1A4500, R4596, and PTXU04 revealed typical characteristics of a podovirus with icosahedral heads and short tails, while the fourth member, WFI101126, showed a more uncommon C3-like morphology with an elongated head (length:width ratio >3) of about 144 nm (Figure 3) as already reported elsewhere [40,41].

### 3.2. Host Spectrum

**Figure 4:**
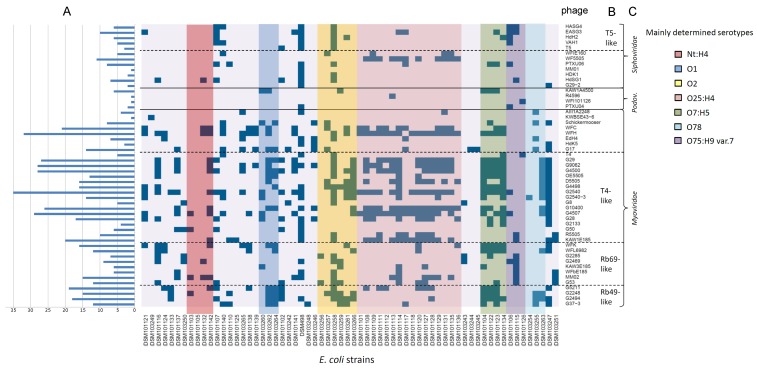
Analysis of the lytic behavior of isolated phages on a set of 64 *Escherichia coli* strains from various serotypes. Blue squares indicate plaque formation of a given phage. The number of strains that were successfully lysed by a given phage are shown in (**A**). Order of the phages (*y*-axis) is based on morphology (**C**) and taxonomic classification (**B**), respectively. The order of *E. coli* strains is based on determined serotypes. As there are many T4-like and T5-like phages in our study, phages T4 and T5 were also included in this analysis.

Altogether, 50 phages were isolated from various habitats, such as surface water, wastewater, manure, or animal feces. The host range of these phages was performed in a qualitative approach by spotting serial dilutions on double agar plates containing potential host cells. Host strains were classified as sensitive when single plaques were generally detected at any dilution. All of them were tested against a set of 64 *E. coli* strains from different origins, e.g., ESBL-producing *E. coli* or avian pathogenic *E. coli*, representing different serotypes. Finally, each *E. coli* strain was lysed by at least one of the phages isolated in this study (Figure 4). Phages showed very diverse lytic behavior, ranging from very specifically lysing only one bacterial strain of this set to infecting and lysing nearly 55% of the tested strains. In particular, members of the *Podoviridae* family revealed a very narrow host range with R4596 and WFI101126 forming plaques only on one specific strain. In contrast, some myoviruses, such as WFH and T4-like phages G2540 and G4507, showed a relatively broad host range and lysed more than 25 strains. Regarding the siphoviruses of this study, those phages also showed a rather narrow host range and lysed only up to 11 strains of the examined set. When analyzing the putative serotype-dependent lysis behavior for those phages that lysed more than 10 strains, no such specificity was detected with the exception of WF5505. While myoviruses with a broader host range lysed strains throughout all serotypes, the siphovirus WF5505 lysed only strains that belonged to the serotype O25:H4.

### 3.3. Genomic Analysis of Isolated Podoviruses

Phage genomes of this study were sequenced using either Illumina or PacBio RS II technology to determine their genome sequence. The assembled sequences were analyzed in particular regarding their relatedness to known phages, taxonomy, and specific features. Generally, phage genome sizes ranged from approximately 71 kb to 371 kb (GC content 35.2–46.5%) for myoviruses, siphoviruses revealed genomes with sizes between approximately 42 and 125 kb (GC content 39.0–54.6%), while the genomes of the isolated podoviruses displayed sizes from approximately 44 up to 77 kb (GC content 42.0–52.85%) (Table S4).

**Figure 5:**
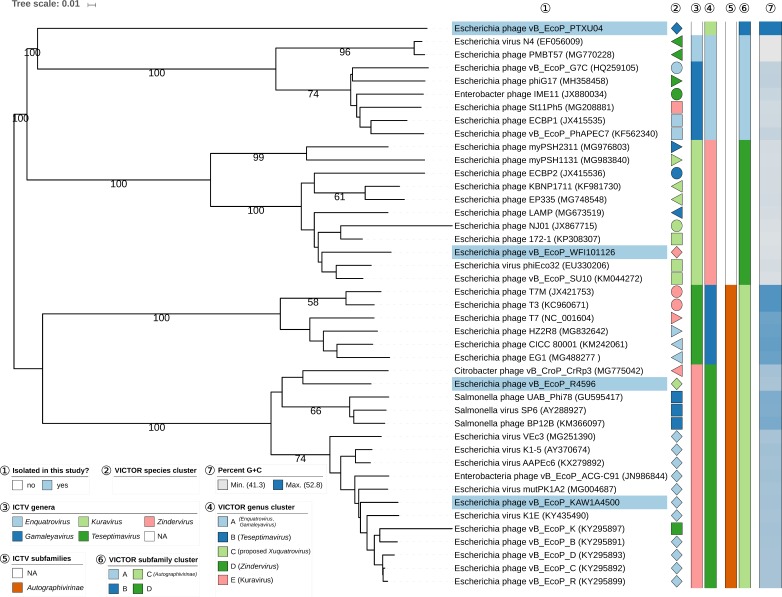
Phylogenomic analysis of *Podoviridae* members of this study at the amino acid level using VICTOR. The recommended VICTOR tree (formula *d_6_*) is shown and the numbers shown above the branches are Genome-BLAST Distance Phylogeny (GBDP) pseudo-bootstrap support values from 100 replications, given that branch support exceeds 50%. The branch lengths of the resulting VICTOR trees are scaled in terms of the used distance formula. Annotations are given to the right-hand side of the tree: indicator for newly isolated phages (1), genera and subfamilies according to International Committee on Taxonomy of Viruses (ICTV) (3,5), species, genus, and subfamily cluster proposed by VICTOR (2,4,6), and genomic G+C content (7).

BLASTn analysis to check for related phage genomes and comparison via dot plot analysis using GEPARD [42] of the genomes of four podoviruses isolated in this study (Figure S1) showed that at least one of them, namely PTXU04, did not reveal any similarities to other phages in databases at the nucleotide level in the BLASTn analysis and was considered as novel. The amino-acid-based VICTOR analysis revealed similarities to other phages only between 0 and 1.44% (see Supplementary Table S5). In contrast, phages KAW1A4500 and R4596 showed VICTOR-based similarities of 73.5% (R4596) and 90.2% (KAW1A4500) to phages K1E (GenBank acc. no. AM084415) and similarities of 73.8% (R4596) and 91.8% (KAW1A4500) to phage vB_EcoP_C (GenBank acc. no. KY295892) and, therefore, were classified as members of the subfamily *Autographivirinae* in the VICTOR analysis, given the established threshold for subfamily delineation [36]. Analysis of the genome of phage WFI101126, which displayed a rare C3-like morphology, revealed that WFI101126 has a similarity of 84.6% (see Supplementary Table S5) to phage phiEco32 (GenBank acc. no. EU330206) and, therefore, belongs to the classified *Kuravirus* (ICTV document 2018.007B.Av1.rename137gen6sp) according to the VICTOR analysis and the established threshold for genus delineation. The results of the VICTOR analysis revealed maximally supported genus clusters and, thus, provided strong support for the proposed creation of a new genus “Xuquatrovirus” within the *Podoviridae* family with phage PTXU04 as the “type phage” (Figure 5). Phages WFI101126, R4596, and KAW1A4500 were placed by VICTOR in maximally supported genus clusters matching known ICTV genera (*Kuravirus*, *Teseptimavirus*, and *Zindervirus*, respectively). Whereas VICTOR placed phages R4596 and KAW1A4500 as members of the *Zindervirus* in the known ICTV subfamily *Autographivirinae*, phages PTXU04 and WFI101126 represent two novel, also maximally supported subfamilies (Figure 5).

### 3.4. Genomic Analysis of Isolated Siphoviruses

Similar to the analysis of the isolated podoviruses, we also performed a BLASTn and subsequent dot plot analysis of the genomes of different siphoviruses with GEPARD (Figure S2). Three phages in our study (WFIE160, PTXU06, and WF5505) showed a high level of synteny compared to, e.g., phages slur05 and EK99P-1 (GenBank acc. nos. LN881730 and KM233151, respectively) amongst others at the nucleotide level and, therefore, were classified to the genus *Dhillonvirus*. Because of their high degree of relatedness to phages T5 or FFH1 (GenBank acc. no. AY543070 and KJ190157, respectively), phages VAH1, EASG3, HASG4, and HdH2 are considered to be new members of the *Tequintavirus*. Two further phages of our study, phages HdSG1 and HdK1, were identified that belong to the *Nonagvirus*, a group of phages like phage 9g [43] or JenP1 (GenBank acc. no. KJ419279 and KP719132, respectively) harboring specific gene clusters for queuosine synthesis. While analysis of phage MM01 grouped it into the *Tunavirinae* subfamily, phage G29-2 showed the highest VICTOR similarity (88.5%; see Supplementary Table S5) to *Shigella* phage pSF-1, which is still unclassified.

**Figure 6:**
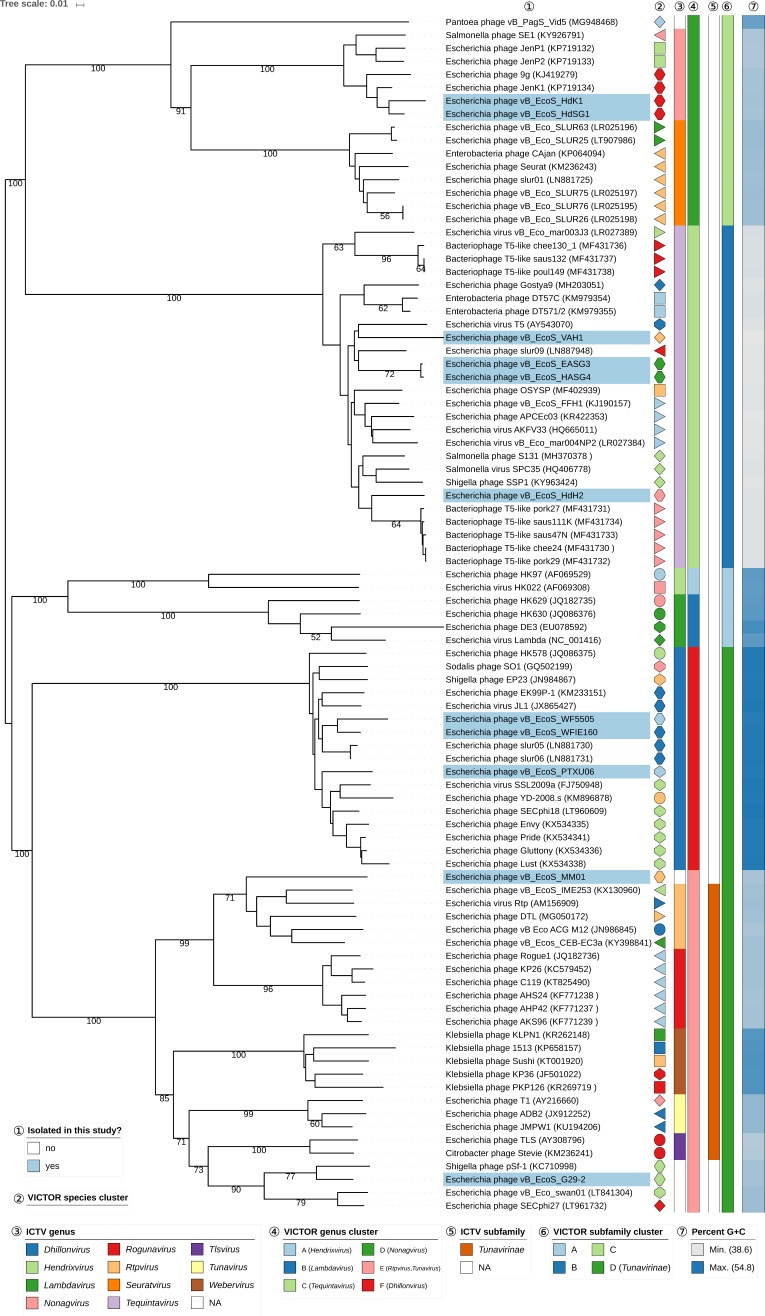
Phylogenomic analysis of *Siphoviridae* members of this study at the amino acid level using VICTOR. The recommended VICTOR tree (formula *d*_6_) is shown and the numbers listed above the branches are GBDP pseudo-bootstrap support values from 100 replications, given that branch support exceeds 50%. The branch lengths of the resulting VICTOR trees are scaled in terms of the used distance formula. Annotations are given to the right-hand side of the tree: indicator for newly isolated phages (1), genera and subfamilies according to ICTV (3,5), species, genus, and subfamily cluster proposed by VICTOR (2,4,6), and genomic G+C content (7).

In regard to the amino-acid-based VICTOR analysis of the siphovirus dataset (Figure 6), all genus clusters were in congruence with the current ICTV genus classification except for genus cluster “E”, which included both *Rtpvirus* and *Tunavirus.* Since VICTOR is optimized against the ICTV classification, this deviation indicates that the existing classification into these two particular genera might be too fine-grained and could be merged into a single ICTV genus as represented by VICTOR cluster “E”, that way including the newly isolated phages MM01 and G29-2. That way, the resulting genera would also be more uniform in terms of sequence divergence, an important criterion of any classification. The classification at the subfamily level revealed that phages MM01, G29-2, WF5505, WFIE160, and PTXU06 can safely be assigned to the *Tunavirinae*, whereas two new subfamilies can be proposed for phages HdK1 and HdSG1 (subfamily cluster “C”) and phages VAH1, HdH2, EASG3, and HASG4 (subfamily cluster “B”).

### 3.5. Genomic Analysis of Isolated Myoviruses

**Figure 7:**
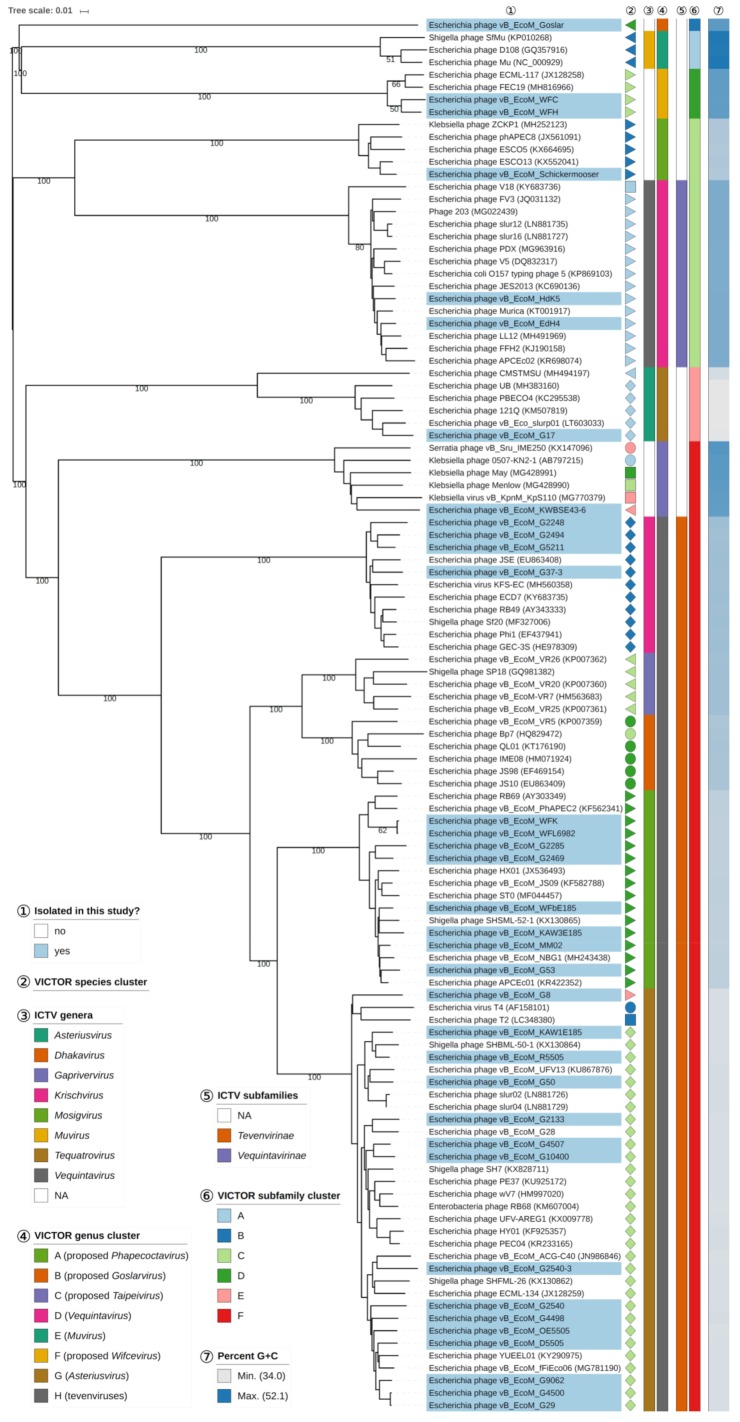
Phylogenomic analysis of *Myoviridae* members of this study at the amino acid level using VICTOR. The recommended VICTOR tree (formula *d*_6_) is shown and the numbers above branches are GBDP pseudo-bootstrap support values from 100 replications, given that branch support exceeds 50%. The branch lengths of the resulting VICTOR trees are scaled in terms of the used distance formula. Annotations are given to the right-hand side of the tree: indicator for newly isolated phages (1), genera and subfamilies according to ICTV (3,5), species, genus, and subfamily cluster proposed by VICTOR (2,4,6), and genomic G+C content (7).

All genomes of the isolated myoviruses of this study were analyzed with BLASTn and GEPARD to visualize nucleotide sequence similarity (Figure S3) complemented by an amino-acid-based VICTOR analysis of the coding regions (Figure 7). Based on those results, the majority of the analyzed myoviruses were placed in the large subfamily *Tevenvirinae* with members distributed over the genera *Krischvirus*, *Gaprivervirus*, *Dhakavirus*, *Mosigvirus*, and *Tequatrovirus*.

Two phages, EdH4 and HdK5, showed high VICTOR similarities (>93%) to *Escherichia* phages Murica and FFH2 (GenBank acc. no. KT001917 and KJ190158, respectively), all of them placed in genus cluster “D” (Figure 7) and, therefore, identified as members of *Vequintavirus*. Other currently unclassified phages were located in distinct VICTOR genus clusters without affiliation to known ICTV genera, i.e., phages Goslar, WFC, WFH, Schickermooser, and KWBSE43-6. Phages WFC and WFH form a species cluster together with phages ECML-117 and FEC19 belonging to a single genus cluster “F” that we tend to call “Wifcevirus”, while phage Schickermooser was classified as a member of the VICTOR genus cluster “A” (proposed “Phapecoctavirus”) Interestingly, phage KWBSE43-6 is phylogenetically most closely related to a maximally supported group of non-*E. coli* phages (genus cluster “C”, proposed “*Taipeivirus*”), mainly with *Klebsiella* as their host genus, and all being members of the *Tevenvirinae* (Figure 7). Phage Goslar is the only myovirus of this study that showed no nucleotide sequence similarity to any known phage after BLASTn analysis. Therefore, we propose to create a new genus within the *Myoviridae*, namely “*Goslarvirus*”. VICTOR strongly supports the proposal of new subfamilies for subfamily clusters “E” (including phage G17), “D” (including phages WFC and WFH), and “B” (phage Goslar).

### 3.6. Genomic Analysis of Genomic Singleton Phages

#### 3.6.1. Phage PTXU04

**Figure 8:**
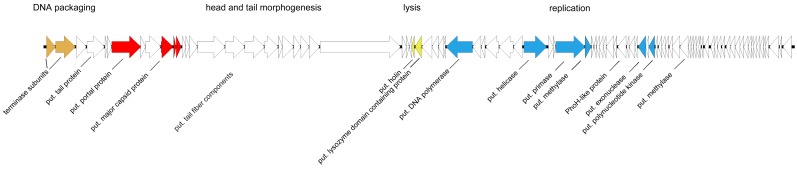
Genomic organization of PTXU04. Genes for identified structural proteins using peptide mass fingerprinting are marked in red, putative genes for DNA packaging in orange, putative genes for lysis in yellow, and genes involved in replication in blue (put. = putative).

Phage PTXU04 revealed a total genome size of 61,600 bp (Figure 8) (GenBank acc. no. MK373772). Time-limited exonuclease treatment of its genomic DNA, followed by hydrolysis with restriction enzymes, also indicated that its genome is not circularly permuted (Figure S4). Automated followed by subsequent manual curated annotation using Prokka and Artemis revealed 92 coding sequences (CDSs), while no tRNA genes were found. Based on conserved domains detected in the deduced amino acid sequences and only weak similarities to annotated genes in GenBank followed by a search against the pVOG database, some essential genes were identified, leading to a grouping of genes into characteristic gene clusters similar to those involved in head and tail morphogenesis, lysis, or replication. Thus, we identified domains, e.g., for the terminase subunit (locus tag PTXU04_00002, PHA02533), a major capsid protein (PTXU04_00010, TIGR04387), a putative NTP-PPase (PTXU04_00033, cd11542), DNA polymerase (PTXU04_00035, cd05538), helicase (PTXU04_00041, COG1061), a primase (PTXU04_00044, TIGR01613), and a putative DNA N-6-adenine-methyltransferase (Dam) (PTXU04_00045, pfam05869).

**Figure 9:**
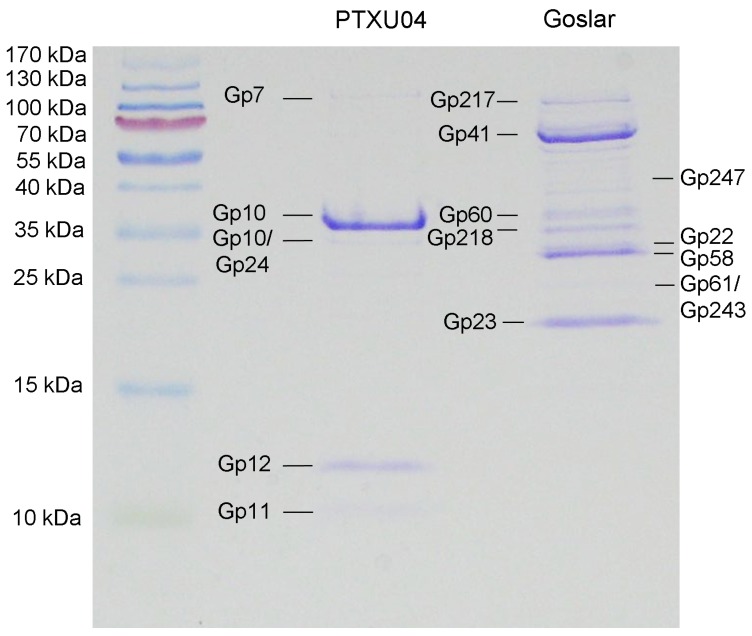
SDS-PAGE profile of structural proteins of phages PTXU04 and Goslar and identification via mass peptide fingerprinting. Proteins were separated by 15% SDS-PAGE. After extraction of gel bands and tryptic digestion, samples were analyzed via peptide mass fingerprinting. Identified gene products are shown.

Since comparison of the genome sequence of PTXU04 with other phages in GenBank via BLASTn (against taxid:28883) resulted in no hits and BLASTp analysis revealed only weak similarities to known phages or conserved gene products, we used mass peptide fingerprinting to obtain hints on the location of some structural genes in the genome and to verify putative annotations. Therefore, phage particles were denatured by boiling and separated by SDS-PAGE (Figure 9). Afterwards, several protein bands were extracted and identified by peptide mass fingerprinting (Table S6). Finally, four bands were clearly assigned to genes of PTXU04, namely *gp7* (PTXU04_00007), *gp10* (PTXU04_00010), *gp11* (PTXU04_00011), and *gp12* (PTXU04_00012) (Figure 8). The most prominent band could be identified as the major capsid protein (*gp10*).

#### 3.6.2. Phage Goslar

**Figure 10:**
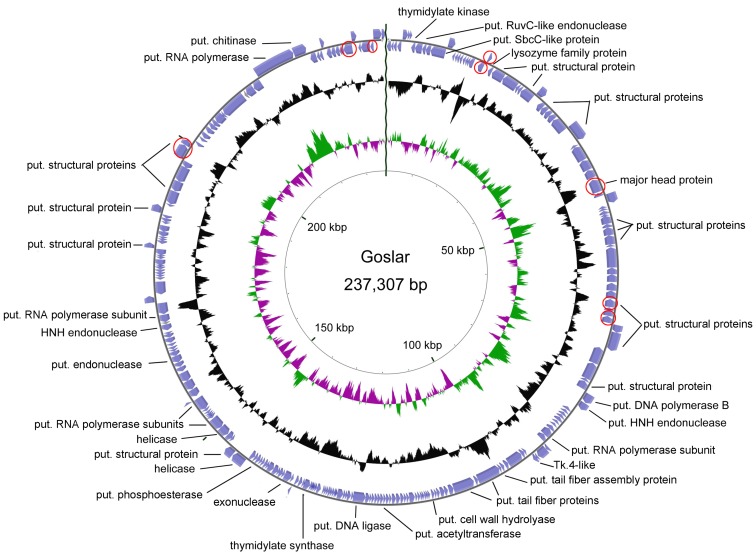
Genomic organization of phage Goslar. Based on its permutation, the genome is visualized in a circular form. Genes for identified structural proteins using peptide mass fingerprinting are marked in red (put. = putative).

Sequencing of phage Goslar resulted in a genome size of 237,307 bp (GC content of 46.53%), classifying it as a jumbo phage. In this case, treatment with exonuclease BAL-31 and subsequent hydrolysis with different restriction enzymes showed that the genome of this phage appears to be circularly permuted. Annotation led to the determination of 249 predicted CDS, but no tRNAs (Figure 10). Though comparison at the nucleotide level resulted in only a small number of short stretches of similarity to other phages, BLASTP analysis gene by gene and hmmscan using the pVOG database revealed several shared conserved domains and similarities to other phages, so that a rough characterization of the genome organization could be performed. Thus, we identified putative genes involved in replication, e.g., a DNA ligase and helicase, in nucleotide metabolism, e.g., a putative thymidylate synthase, and a chitinase, which may be involved in host lysis. Based on the number of hits in the BLASTP analysis, we chose phages from GenBank for further analysis with CoreGenes3.5 [44,45]. Thus, we identified non-*Escherichia* phages that shared at least more than 70 homologs with phage Goslar, namely *Pseudomonas* phages phiKZ (77), PhiPA3 (79), Phabio (77), and Noxifer (84), *Erwinia* phages vB_EamM_ChrisDB (76), PhiEAH1 (83), and Ea35-70 (91), and *Ralstonia* phage RSL2 (75).

In addition, peptide fingerprinting analysis (Figure 9, Table S6) identified 10 genes for structural components that are not located in one cluster, but distributed over the whole genome of Goslar, including the gene for a major capsid protein (Goslar_00041).

### 3.7. Grouping of Isolated Phages into Already Classified Genera

#### 3.7.1. Phieco32-Like Viruses

**Figure 11:**
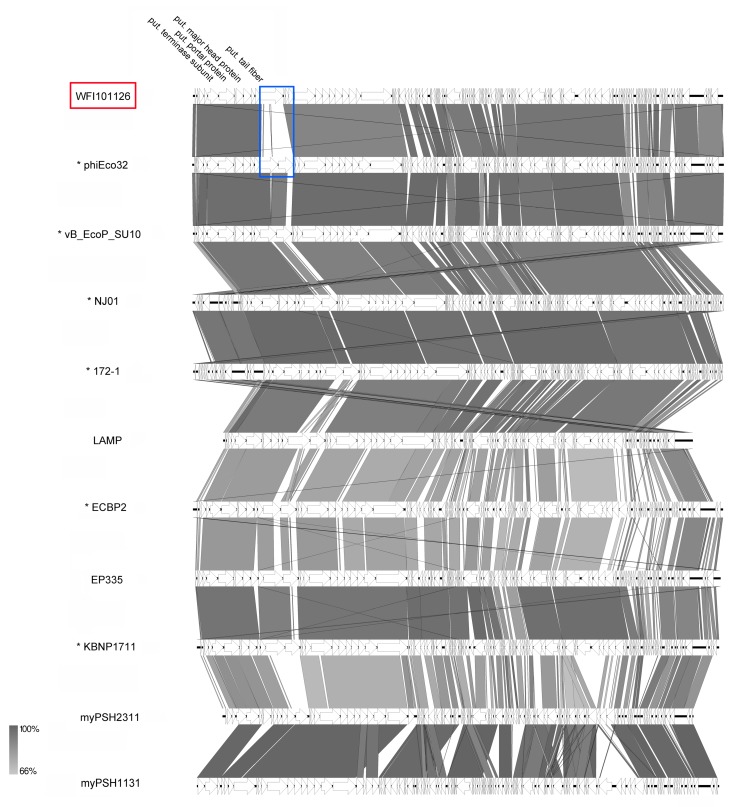
Synteny plot of phage WFI101126 compared to other kuraviruses at the nucleotide level (pairwise comparison with the genome next to it on the figure). The figure was generated with EasyFig [48]. Asterisks indicate official classification as the genus *Kuravirus is* already recognized by ICTV. Isolates of this study are marked in red.

Based on their striking C3-like morphological features displaying elongated capsids at first hand and also on genomic and proteomic approaches, the genus *Kuravirus* was created by the ICTV and consists currently of six officially classified members (Figure 9), including already published phages phiEco32 [41], vB_EcoP_SU10 [40], NJ01 [46], and ECBP2 [47]. Generally, after our analysis, all suggested members had a genome size of about 77 kb (average GC content 42.2%), encoded about 74 proteins, and revealed 0-1 tRNAs with the exception of phages LAMP (MG673519) and myPSH2311 (MG976803), which have smaller genomes of about 68.6 kb. Overall, the ends of the WFI101126 genome were adjusted based on PhiEco32 and vB_EcoP_SU10, which revealed the strongest similarities (VICTOR similarity of 92.1%; see Supplementary Table S5). A synteny plot of different kuraviruses (Figure 11) showed that they all have nearly the same genome organization apart from small differences. One major difference of WFI101126 is the organization of the cluster for putative tail fibers. While all other phiEco32-like phages seem to have several genes encoding tail-fiber-associated proteins, WFI101126 seems to harbor one less (WFI101126_0001212, marked in blue). Altogether, our analysis of WFI101126 isolated in this study compared to other related phages clearly classified it and four other phages as members of this distinct genus.

#### 3.7.2. Sp6-Like Viruses

**Figure 12:**
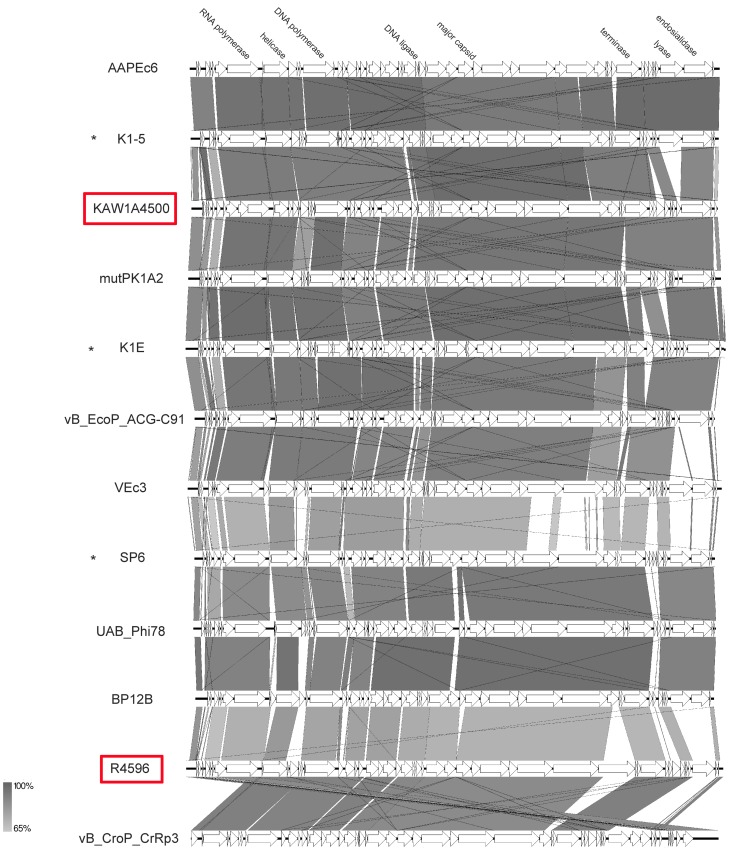
Genomic organization of phages R4596 and KAW1A4500 compared to selected zinderviruses at the nucleotide level. The figure was generated with EasyFig [48]. Asterisks indicate official classification as *Zindervirus* already ratified by ICTV. Isolates of this study are marked in red.

In this study, we isolated two podoviruses that revealed icosahedral heads and short tails. After sequencing and genome analysis, they were found to be similar to SP6 [49], two capsular-specific phages [50], and a set of other *E. coli* phages from a historic collection [51] with respect to their genome organization. Genome sizes ranged from 37.7 to 45.2 kb with an average GC content of 45.5%. As with other SP6-like phages, none harbored a tRNA gene. As described for some SP6-like phages [51], we also detected short terminal repeats of 266 bp and 428 bp for KAW1A4500 and R4596, respectively. While phage KAW1A4500 was similar to capsular-specific phages K1E and K1-5 (VICTOR similarity of c. 90%; see Supplementary Table S5) and other *Escherichia* phages of that genus, R4596 tends to cluster with phages with *Salmonella* as their host organism, such as phage UAB_Phi78 (Figure 10). Though very similarly organized, their genome structure revealed some differences, in particular in the gene cluster for enzymes putatively involved in lysis (Figure 12). The gene cluster identified in KAW1A4500 seems to be conserved among its closest relatives apart from an additional gene (KAW1A4500_00051), which was only identified in phage mutPK1A2 (mutPK1A2_p*54*) as well. Interestingly, phages K1-5 and AAPEc6 additionally harbored a gene encoding a putative lyase (conserved domain Peptidase_S74 pfam13884) separated from the conserved endosialidase gene (conserved domains pfam12217–19). In comparison, *Salmonella* phage UAB_Phi78, SP6, and BP12B possessed two genes in this region, one encoding a P22-like tail-spike (pfam09251). Analysis of this region in the genome of phage R4596 showed that it carried an additional gene (R4596rev_000*38*) upstream in the structural gene cluster that does not appear in all other genomes. Its gene product revealed a conserved domain (pfam03906) for a T7-like tail fiber structure. Apart from that, R4596 did not show any sign of an endosialidase gene, but one gene for a putative pectate lyase (pfam12708) (R4596rev_00049).

#### 3.7.3. T5-Like Viruses

**Figure 13:**
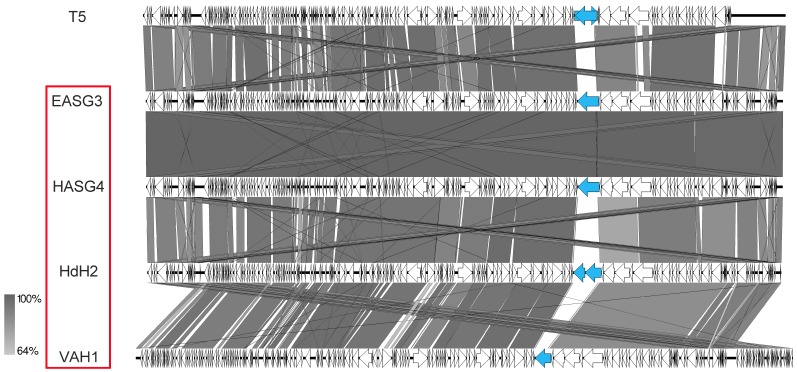
Genomic organization of T5-like phages of this study compared to phage T5 at the nucleotide level. The figure was generated with EasyFig. Isolates of this study are marked in red.

Several new T5-like phages have been isolated from different habitats and studied in recent studies [19,20]. In our study, we also identified four phages closely related to *Escherichia* phage T5. All of them were isolated from samples associated with different birds. While phages HdH2 and HASG4 were identified using chicken feces, phages EASG3 and VAH1 were isolated from duck and budgie droppings, respectively. In regard to their sequence, the genome sizes ranged between 120 and 124 kb, displaying the characteristic T5-like terminal repeats of about 10–11 kb and 21–24 tRNA genes. Major differences were again detected in the region for genes involved in adsorption, in particular in the gene for the L-shaped tail fiber of T5, which is exchanged in the other phages (Figure 13, marked in blue). While the homolog in EASG3 and HASG4 revealed similarities to a tail fiber gene from *Salmonella* phage SP1a, HdH2 harbored two genes for tail-fiber-like proteins similar to *Salmonella* phage Sw2. Further differences were also identified in the gene for the T5 straight tail fiber (T5.140) and the receptor binding protein (T5.157), which might be the reason for different host ranges of the T5-like phages of our study.

#### 3.7.4. 9g-Like Viruses

**Figure 14:**
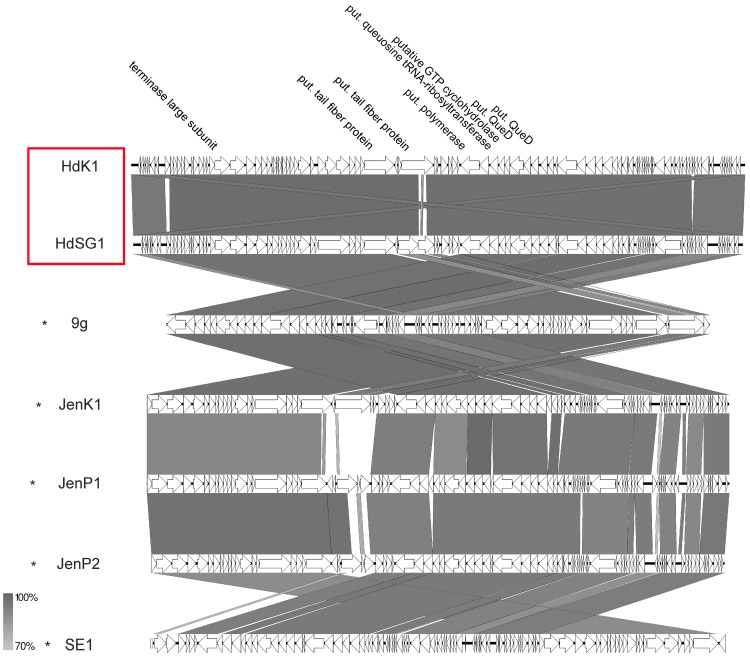
Synteny plot of phages HdK1 and HdSG1 compared to phage 9g at the nucleotide level. The figure was generated with EasyFig. Asterisks indicate official classification as members of the ICTV-recognized genus *Nonagvirus*. Isolates of this study are marked in red.

Two siphoviruses of our study, namely HdK1 and HdSG1, that were isolated from chicken dung revealed high VICTOR similarities >86.4% (see Supplementary Table S5) to phage 9g and phages belonging to the *Nonagvirus* genus. However, a few differences were detected. As already described for other phage groups of this study, those differences were mainly detected in the cluster for putative tail fibers or other gene products involved in adsorption (Figure 14). So far, five similar phages (*Salmonella* phage SE1 and *Escherichia* phages 9g, Jenk1, JenP1, and JenP2) isolated from fecal matters are described in the literature displaying the same genomic organization as HDK1 or HdSG1 [43,52]. Altogether, their genome sizes range from 56.7 to 64 kb with an average GC content of 43.6%. The main identifiable characteristic of the genera *Nonagvirus* and *Seuratvirus* is the presence of a queuosine synthesis cluster [53,54]. In phage HdK1, this cluster contains genes for a putative queuosine tRNA-ribosyltransferase similar to that of phage 9g (HdK1rev_000*55*), a putative queuosine biosynthesis protein FolE (conserved domain FolE COG0302 GTP cyclohydrolase I) (HdK1rev_000*56*), a putative 6-carboxy-5,6,7,8-tetrahydropterin synthase (conserved domain queuosine_QueD TIGR03367) (HdK1rev_000*57*), a putative queuosine biosynthesis QueC ATPase (conserved domain QueC COG0603 7-cyano-7-deazaguanine synthase) (HdK1rev_000*58*), and a putative queuosine biosynthesis QueE (conserved domain NrdG COG0602) (HdK1rev_000*60*) identical to *Enterobacteria* phage JenK1, flanked by genes for a helicase and DNA polymerase, respectively. Some similar examples of such a gene cluster have also already been reported for phages with other hosts than *E. coli*, e.g., *Mycobacterium* phage Rosebush [55], *Streptococcus* phage Dp-1 [56], or *Pantoea* phage vB_PagS_Vid5 [57]. The described gene cluster might be responsible for the modification of DNA to probably provide resistance against different restriction enzymes. As also described for phage 9g [43], phage HdK1 is resistant to some of the tested restriction enzymes (data not shown) which might be a hint that modified bases also exist in the genome of HdK1.

#### 3.7.5. HK578-Like Viruses

**Figure 15:**
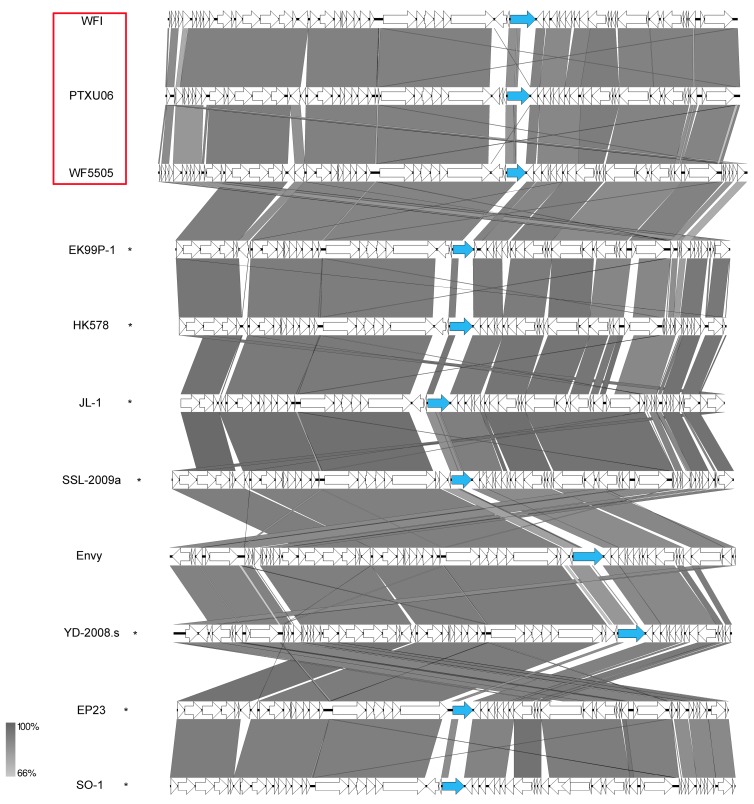
Synteny plot of phages WFIE160, WF5505, and PTXU06 and an overview of the genomic organization of selected HK578-like phages at the nucleotide level. The figure was generated with EasyFig. Asterisks indicate ICTV-classified species within the genus as *Dhillonvirus*. Isolates of this study are marked in red.

A further group of siphoviruses isolated in this study was clustered into the ICTV-classified genus *Dhillonvirus* based on their morphological similarities, revealing quite flexible tails and genomic organization. Further members of this group, such as phages slur05 and slur06, phage EP23, or phage JL1, have already been described in the literature [58,59,60]. They all show nearly the same gene content with small variations (Figure 15). Our phages revealed genome sizes of about 42–47 kb with a GC content of about 54.4% and carried no tRNA genes. Interestingly, WFI, WF5505, and PTXU06 differ in particular in one gene (WF5505 *gp38*) for a tail fiber (Figure 13, marked in blue), which might correlate to the distinct differences of our isolated phages in regard to their host spectrum and might make WF5505 specific for serotype O25:H4.

#### 3.7.6. Rtp-Like Viruses

A further siphovirus of our study was classified as a new member of the *Tunavirinae* subfamily. The first sequence analysis showed that MM01, which was isolated from horse dung, had the same genomic organization as other members of the *Tunavirinae* subfamily with rtpviruses as its closest relatives (Supplementary Figure S5) [61,62,63,64]. The rtpviruses revealed an average genome size of 45.8 kb with an average GC content of 44.2% and carried one single tRNA, while the genome size of phage MM01 is a little bit smaller (43.2 kb). Variations in gene content were observed again in the cluster for tail fiber proteins. Generally, those genes were identified at different locations in the genome. While one gene is usually embedded in the cluster for other structural components, e.g., minor tail proteins or a tail tape measure protein, another gene for a tail fiber component is located on the other strand. Interestingly, phage MM01 might harbor three genes for putative tail fibers, namely MM01_00019, MM01_000*36*, and MM01_000*44*, though the real function of MM01_00019 is not clear yet. While MM01_00036 is quite similar to its homolog in phage RTP, Gp44 shows similarities to a tail fiber protein of phage KBNP1711. Electron microscopy revealed that MM01 has a flexible tail of about 153 nm and a head length of about 64 nm. Similar studies with phage RTP showed that it has a unique tail tip consisting of four leaf-like structures arranged in a rosette [65].

#### 3.7.7. pSf1-Like Viruses

Phage G29-2 was isolated from manure in Germany and showed characteristic features of members of the *Siphoviridae* family. It has a flexible tail with a length of about 143 nm and an icosahedral head with a length of about 69 nm. Sequence comparison and phylogenomic analysis via VICTOR unveiled that, together with its closest relative *Shigella* phage pSf-1, G29-2 can be classified into the *Tunavirus*. pSf-1 was also reported to display a quite flexible tail with a length of 103 nm and a head diameter of 73 nm [66]. Both phages revealed a genome size of about 51 kb with a GC content of 44%, showing no signs of tRNAs.

#### 3.7.8. T4-Like Viruses

After analysis of the myoviruses, 27 of our isolates were classified as new members of the *Tevenvirinae* subfamily, including the three genera, namely *Tequatrovirus*, *Mosigvirus*, and *Krischvirus*. Revealing morphological features undistinguishable from phage T4, only a genomic analysis of those isolates provided further insights into their grade of relatedness (Figure 7). Originally, all those different genera belonged to the *T4likevirus*, but were separated into different genera by the ICTV based on different features. Phylogenetic analysis of phage RB49 revealed a distance from T4 and justified a classification as a pseudo-T-even phage [67]. Some members of this genus (*Krischvirus*) have also been described in literature dealing with different topics [68,69,70,71]; they have a slightly smaller genome size (~164 kb) than T4-likes, carry no tRNAs, and their DNA contains cytosine and no 5-hydroxymethylcytosine. In comparison, RB69-like phages are much more closely related to T4 at the nucleotide level than krischviruses; most of the members, such as RB69 or PhaPEC2 [72,73], have a genome size of about 168 kb, similar to T4, and reveal two or three tRNAs while tequatroviruses of our study displayed up to 12 tRNAs. Though our isolated phages belonging to those three genera are quite similar, in fact forming a single genus cluster “E” in the VICTOR analysis (Figure 7), showing only small variations in gene content, and being, therefore, considered as new isolates, but not new species, their lytic behavior on different *E. coli* strains (Figure 4) showed distinct differences. Interestingly, we identified some variations in particular in *gp37*- and *gp38*-likes of T4, encoding putative components of a tail fiber.

Here, we have uncovered many nearly identical tequatroviruses, as evidenced by the sequence similarities of their structural protein genes and electron microscopy images. An interesting aspect of new phage discovery is their host specificity, which is determined to a great extent by their fiber protein structure [74]. Due to horizontal gene transfer, sequence similarity of the tail fibers, especially those of the receptor-binding C-termini, often does not show correlation with the rest of the structural proteins. The C-terminal ends of the translated gp37 genes of phages MM02, KAW3E185, WFbE185, G2285, G53, KAW1E185, R5505, and G2469 all have between seven and nine repeats of the putative iron ion coordinating His-X-His sequence motifs at their C-terminal ends. This means that the long tail fiber tips of these phages are likely to have a similar structure to the gp37 needle of T4 (PDB 2XGF; [75]). This is confirmed by HHpred analysis [76].

The C-terminal ends of the translated gp37 genes of phages G10400 and G50 are predicted to be structurally similar to the C-terminal end of gp37 of Salmonella phage S16 (Protein Data Bank (PDB) 6F45, [77]). For phage S16, gp38 stays bound to gp37 and is probably the de facto receptor-binding protein. Indeed, for phages G10400 and G50, the gp38 genes also show predicted structural similarity to gp38 from S16.

The other new gp37 sequences are not predicted to have structural similarity to gp37 of phage T4 or S16. These are interesting candidates for future structural studies. Previously known tequatrovirus gp37 sequences also show predicted structural similarity to gp37 of T4 or S16, or fall in the unknown category. For example, the *E. coli* phage T2 gp37 is predicted to have structural similarity to the *Salmonella* phage S16.

#### 3.7.9. V5-Like Viruses

One further group of larger myoviruses was classified as *Vequintavirus* belonging to the subfamily *Vequintavirinae*. This subfamily consists of four genera *Avunavirus* (1 sp.; 121 kb, 40.0% G+C, 17 tRNAs); *Certrevirus* (5 sp.; 150 kb, 51 mol% G+C, 17 tRNAs); *Seunavirus* (4 sp; 148 kb, 46% G+C, 24 tRNAs); and *Vequintavirus* (8 sp.; 138 kb, 44 mol% G+C, 5 tRNAs). The type phage for that group, V5, was used in a set of phages for typing of Shiga-toxin-producing *E. coli* O157:H7. At least further seven vequintaviruses have been described in the literature before [58,78,79,80,81,82,83]; most of them had *E. coli* O157:H7 as their host. Two of our isolated phages, EdH4 and HdK5, revealed characteristic features of this group and, thus, were included in that genus (Supplementary Figure S6). Both have short terminal redundancies of 458 bp, a genome size of 136,031 bp and 139,328 bp, respectively, and carry seven tRNAs.

#### 3.7.10. PBECO4-Like Viruses

Apart from already described phages of this study with “regular” well-known genome sizes, such as, for instance, for tequatroviruses, we also isolated a phage, G17, which revealed a genome size of more than 370 kb. Such phages with genome sizes >200 kb are classified as “jumbo phages”. Due to the rising number of genome sequences, more and more of those have been recently identified to originate from various habitats and infect different host genera [84]. The most famous and revealing the largest genome so far is *Bacillus megaterium* phage G [85]. Further well-known jumbo phages infecting other genera are, for instance, *Klebsiella* phage vB_KleM-Rak2 [86], *Cronobacter* phage GAP32 [87], and *Pseudomonas* phage ϕKZ [88]. Analysis of the genome of phage G17 revealed 659 CDS and seven tRNAs; sequence coverage showed large terminal redundancies of about 20 kb. Comparison with the GenBank database for tailed phages (taxid:28883) showed high similarities (VICTOR similarities >89) to phages 121Q, slurp01 [89], and PBECO4, underlined by similarities in gene content and genome organization (Supplementary Figure S7). Apart from its large genome, its morphology displayed large features with a head length of about 139 nm and a head width of about 129 nm (Table 1). Recently, the ICTV proposed to create a new genus, *Asteriusvirus*, derived from Greek mythology for those gigantic *E. coli* phages related to PBECO4 isolated in Korea [90]. Thus, G17 is considered to be a member of this new genus.

### 3.8. Proposal of Newly Created Genera

During our analysis, we recognized some phages that, according to their sequence similarity, were considered either as completely novel, e.g., PTXU04, or as a new member of a yet to be classified genus.

#### 3.8.1. 0507-KN2-1-Like Viruses

Myovirus KWBSE43-6 was isolated from sewage in Braunschweig, Germany, and revealed a highly specific behavior in our host range analysis, lysing only one specific *E. coli* strain of our set. BLASTn analysis revealed that its nucleotide sequence was similar to five other unclassified phages, mainly *Klebsiella* phages, with VICTOR similarity values between 80 and 85%, forming a single VICTOR genus cluster (Figure 7). Only two phages of this new genus have been published so far. Besides *Serratia* phage vB_Sru-IME250 [91], only the first isolate of this group, *Klebsiella* phage 0507-KN2-1, was further analyzed in particular in regard to its polysaccharide depolymerase [92]. All of the included phages revealed genome sizes between 154 and 160 kb, an average GC content of about 46.8%, and four to seven tRNAs. Based on a synteny plot (Supplementary Figure S8) of all those phages, the main variations were again identified in the putative cluster for adsorption and receptor binding. Gp148 of KWBSE43-6 displayed a conserved domain for a putative polygalacturonase (Pgu1 COG5434). Deduced amino acid sequences of following genes downstream were partially similar to annotated tailspike proteins though did not reveal any conserved domains. As KWBSE43-6 lysed only one *E. coli* strain and showed distinct similarities to *Klebsiella* phages at the sequence level, we also tested this phage on four *Klebsiella oxytoca* and 15 *Klebsiella pneumoniae* strains (Table S2). However, no successful lysis by emerging plaques was detected on those strains. Based on the analyzed sequence similarities, we intend to create a new genus “*Taipeivirus*” originating from the first isolate, phage 0507-KN2-1, which was isolated from sewage in Taipei.

#### 3.8.2. ECML-117-Like Viruses

During genome analysis of phages WFC and WFH, we determined strong similarities to only two other phage genomes in GenBank at the nucleotide level, namely phage ECML-117 and FEC19 (VICTOR similarity values >90%, forming a single genus cluster) (Supplementary Figure S9). Sequencing of WFC and WFH resulted in contigs with a final size of 72,472 and 71,283 bp, respectively, including a terminal repeat of about 3.7 kb each. None of the analyzed phages revealed tRNAs in their genome. Based on phage WFC, we intend to create a new genus “*Wifcevirus*”.

#### 3.8.3. phAPEC8-Like Viruses

According to the VICTOR analysis (Figure 7), phage Schickermooser was identified as a new strain of phage ESCO13, which was isolated in France in 2014. Together with phages ESCO5, ZCKP1, and phAPEC8, they form a maximally supported and yet unclassified cluster in the phylogenomic analysis of the *Myoviridae family* (Figure 7). All of them display genome sizes between 147 and 151 kb with an average GC content of 39%. For phage Schickermooser, we also identified a terminal repeat of 328 bp. Additionally, 10–13 tRNAs and a conserved genome structure (Supplementary Figure S10) were detected in all members of that group. Apart from phage Schickermooser, some morphological features were also described in the literature for phage ZCKP1 [93]. Electron microscopy revealed that ZCKP1 had typical characteristics of a myovirus with an icosahedral head and a contractile tail. It has a head size of about 80 nm and a tail length of about 138 nm, while the tail of phage Schickermooser is shorter. In contrast to the phages of our study, ZCKP1 was isolated on a *Klebsiella pneumoniae* strain in Egypt. Host range analysis of that phage revealed that it was capable of infecting and lysing various strains of different members of the *Enterobacteriaceae*-like *K. pneumoniae*, *Proteus mirabilis*, and *E. coli*. Based on phage phAPEC8, which was described as the first member of this new genus [94], we intend to call this new genus “*Phapecoctavirus*”.

## 4. Conclusions

Classification and taxonomy of bacterial viruses is the responsibility of the International Committee in the Taxonomy of Viruses (ICTV). This classification was mainly based on morphological studies [95,96] and properties of the hereditary material until the current era of sequencing, and its new nucleotide sequencing technologies have led to vast numbers of new, yet unclassified, phage genomes being entered into public databases every year [17]. Deeper genomic analyses of some of these data have led to the creation of a fourth family in the order *Caudovirales*, including different genera of myoviruses, and hence revealing the limitations of a solely morphology-based classification and calling for more accurate methods (e.g., sequence-based approaches). Based on the now available data, different approaches and tools for genome- and proteome-based taxonomy have been developed [36,97,98,99] and discussed by the ICTV. Currently, there are 410 ratified genera and 28 families. However, due to the still rapidly rising number of available phage genomes, many phage genomes still remain unclassified. To that end, VICTOR [36], a modern phylogeny-driven classification, was recently developed and yields phylogenies with high agreement with the ICTV classification, including the majority of taxa being well-supported and monophyletic. This approach is fast and was even applied to a large dataset of more than 4000 unclassified phage sequences from GenBank [36]. In our study, VICTOR supported the proposal of seven new genera, thus improving the taxonomy of *E. coli* phages, in particular. Apart from the taxonomic point of view, this study also shows that though having been already intensively studied for decades, novel phages against *E. coli* can still be isolated that show no similarities to known *Escherichia* phages in databases at the nucleotide level at all. These so far unknown sequences offer new possibilities for different aspects of phage research apart from taxonomy. Analysis of their genomes might give insight into different mechanisms of phage–host interaction and replication as the overall genome composition and organization might vary from those that are already known. In regard to future applications of phages or phage components, completely novel phage genomes might also act as sources for identification of new mechanisms for host takeover or enzymes that might help to fight antimicrobial resistance in the future. Our analysis of tail fibers showed that, though we isolated phages that revealed a very high similarity at the nucleotide level, only small genomic changes might vary the host spectrum. Thus, it is still worth looking for new phages to obtain more insight into the diverse phage biology and to enlarge the number of alternatives to fight antimicrobial resistance (AMR) infections.

## Figures and Tables

**Table 1:** Morphological analysis of isolated phages.

Morphotype	Phage	Head Width (nm)	Head Length (nm)	Tail Length (nm)	Classification Based on Head Length/Width Ratio
Siphoviruses	vB_EcoS_HASG4	76 ± 2	79 ± 3	183 ± 19	B1
	vB_EcoS_EASG3	79 ± 3	80 ± 7	199 ± 39	B1
	vB_EcoS_HdH2	76 ± 2	79 ± 3	199 ± 11	B1
	vB_EcoS_VAH1	72 ± 9	79 ± 5	193 ± 45	B1
	vB_EcoS_WFIE160	61 ± 1	64 ± 3	155 ± 23	B1
	vB_EcoS_WF5505	60 ± 1	61 ± 3	136 ± 10	B1
	vB_EcoS_PTXU06	59 ± 2	64 ± 2	141 ± 7	B1
	vB_EcoS_MM01	61 ± 2	64 ± 3	153 ± 5	B1
	vB_EcoS_HDK1	58 ± 4	72 ± 2	223 ± 5	B2
	vB_EcoS_HdSG1	62 ± 3	79 ± 3	182 ± 4	B2
	vB_EcoS_G29-2	66 ± 3	69 ± 3	143 ± 10	B1
Podoviruses	vB_EcoS_KAW1A4500	56 ± 2	58 ± 1	10 ± 1	C1
	vB_EcoP_R4596	60 ± 2	60 ± 3	11 ± 1	C1
	vB_EcoP_WFI101126	47 ± 2	144 ± 3	16 ± 3	C3
	vB_EcoP_PTXU04	79 ± 1	76 ± 2	14 ± 1	C1
Myoviruses	vB_EcoM_Goslar	124 ± 6	123 ± 7	213 ± 9	A1
	vB_EcoM_KWBSE43-6	85 ± 5	93 ± 4	108 ± 4	A1
	vB_EcoM_Schickermooser	82 ± 1	88 ± 11	102 ± 9	A1
	vB_EcoM_WFC	73 ± 3	79 ± 5	110 ± 4	A1
	vB_EcoM_WFH	71 ± 4	79 ± 4	111 ± 2	A1
	vB_EcoM_EdH4	79 ± 5	81 ± 6	112 ± 1	A1
	vB_EcoM_HdK5	80 ± 4	88 ± 4	108 ± 2	A1
	vB_EcoM_G17	129 ± 12	139 ± 4	117 ± 4	A1
	vB_EcoM_G29	91 ± 4	120 ± 7	110 ± 2	A2
	vB_EcoM_G9062	89 ± 2	118 ± 6	106 ± 4	A2
	vB_EcoM_G4500	90 ± 4	117 ± 5	107 ± 2	A2
	vB_EcoM_OE5505	79 ± 4	106 ± 5	104 ± 6	A2
	vB_EcoM_D5505	87 ± 7	108 ± 5	102 ± 7	A2
	vB_EcoM_G4498	87 ± 4	112 ± 3	105 ± 3	A2
	vB_EcoM_G2540	91 ± 3	119 ± 3	110 ± 3	A2
	vB_EcoM_G2540-3	82 ± 4	112 ± 3	110 ± 3	A2
	vB_EcoM_G8	86 ± 6	115 ± 4	104 ± 2	A2
	vB_EcoM_G10400	84 ± 6	116 ± 4	109 ± 3	A2
	vB_EcoM_G4507	82 ± 5	114 ± 6	107 ± 4	A2
	vB_EcoM_G2133	85 ± 4	114 ± 5	105 ± 4	A2
	vB_EcoM_G50	86 ± 7	114 ± 3	100 ± 6	A2
	vB_EcoM_R5505	83 ± 4	105 ± 5	104 ± 6	A2
	vB_EcoM_KAW1E185	84 ± 5	116 ± 4	107 ± 4	A2
	vB_EcoM_WFK	85 ± 4	112 ± 4	104 ± 9	A2
	vB_EcoM_WFL6982	86 ± 7	113 ± 5	106 ± 2	A2
	vB_EcoM_G2285	84 ± 5	114 ± 7	111 ± 5	A2
	vB_EcoM_G2469	88 ± 4	111 ± 6	108 ± 3	A2
	vB_EcoM_KAW3E185	86 ± 2	113 ± 4	99 ± 8	A2
	vB_EcoM_WFbE185	85 ± 5	116 ± 8	104 ± 3	A2
	vB_EcoM_MM02	90 ± 6	112 ± 5	108 ± 8	A2
	vB_EcoM_G53	85 ± 1	109 ± 12	102 ± 8	A2
	vB_EcoM_G5211	84 ± 3	114 ± 3	110 ± 6	A2
	vB_EcoM_G2248	80 ± 2	109 ± 8	104 ± 4	A2
	vB_EcoM_G2494	82 ± 2	115 ± 1	113 ± 4	A2
	vB_EcoM_G37-3	78 ± 3	108 ± 2	110 ± 3	A2

## Supplementary Materials

The following are available online at https://www.mdpi.com/1999-4915/11/5/454/s1, Table S1: *E. coli* strains for phage isolation, characterization, and propagation, Table S2: Klebsiella strains and growth conditions used for host spectrum analyses of phages KWBSE43-6 and Goslar, Table S3: Additional information on phages isolated in this study, Table S4: Morphological and genomic features of isolated phages of this study, Table S5: Pairwise VICTOR intergenomic sitance and similarity data on isolated phages, Table S6: Mascot research results for peptide fingerprinting analysis of structural proteins of phages PTXU04 and Goslar, Figure S1: Dot plot analysis of genomes of isolated podoviruses of this study compared to related genomes and further members of different genera of the *Podoviridae* family, Figure S2: Dot plot analysis of genomes of isolated siphoviruses of this study compared to related genomes and further members of different genera of the *Siphoviridae* family, Figure S3: Dot plot analysis of genomes of isolated myoviruses of this study compared to related genomes and further members of different genera of the *Myoviridae* family, Figure S4: Time-limited digestion of genomic DNA of phage PTXU04 with BAL 31, followed by complete hydrolysis with BglI, Figure S5: Synteny plot of phage MM01 compared to related phages of the rtpviruses and phage T1 at the nucleotide level, Figure S6: Genomic organization of phages EdH4 and Hdk5 compared to related viruses at the nucleotide level, Figure S7: Synteny plot of phage G17 compared to related asteriusviruses at the nucleotide level, Figure S8: Genome organization of phage KWBSE43–6 compared to related phages at the nucleotide level, Figure S9: Synteny plot of phage WFC and WFH compared to related phages at the nucleotide level, Figure S10: Synteny plot of phage Schickermooser compared to related phages at the nucleotide level.
