# Supplementary material for: Still Something to Discover: Novel Insights into Escherichia coli Phage Diversity and Taxonomy
Source: Viruses. 2019 May 17;11(5):454. doi: 10.3390/v11050454 (PMC6563267; doi:10.3390/v11050454)
Supplement: Supplementary file 1 [file viruses-11-00454-s001.zip › viruses-474118-supplementary- Table S1-4, S6 and Figure S1-10.pdf]

Table S1. *E. coli* strains for phage isolation, characterization and propagation

strain designation	origin	serotype	remarks
DSM 613 (B)	ND	O7:H^{NT}	laboratory strain, risk group 1
DSM 498 (K-12)	ND	O16:H48	laboratory strain, risk group 1
DSM 101101	human urine	O25:H4	ESBL, risk group 2
DSM 101102	human	O102:H^{NT}	ESBL, risk group 2
DSM 101103	human	O^{NT}:H4	ESBL, risk group 2
DSM 101104	human urine	O7:H5	ESBL, risk group 2
DSM 101105	human	O^{NT}:H4	ESBL, risk group 2
DSM 101106	human urine	O75:H9 var. 7	ESBL, risk group 2
DSM 101107	human urine	O^{rough}:H6	ESBL, risk group 2
DSM 101108	human urine	O25:H4	ESBL, risk group 2
DSM 101109	human urine	O25:H4	ESBL, risk group 2
DSM 101110	human	O^{NT}:H9 var. 7	ESBL, risk group 2
DSM 101111	human	O25:H4	ESBL *blaCTX-M1*, *-M15*, risk group 2
DSM 101112	human	O25:H4	ESBL *blaCTX-M1*, *-M15*, risk group 2
DSM 101113	human	O25:H4	ESBL *blaCTX-M1*, *-M15*, *blaTEM*, risk group 2
DSM 101114	human	O25:H4	ESBL *blaCTX-M1*, *-M15*, *blaTEM*, risk group 2
DSM 101115	human	O75:H9 var. 7	ESBL, risk group 2
DSM 101116	human	O^{NT}:H15	ESBL, risk group 2
DSM 101117	human urine	O25:H4	ESBL, risk group 2
DSM 101118	human	O25:H4	ESBL, risk group 2
DSM 101120	human	O25:H4	ESBL *blaCTX-M1*, *-M15*, *blaTEM*, risk group 2
DSM 101121	human	O^{NT}:H1	ESBL *blaCTX-M1*, *-M15*, *blaTEM*, risk group 2
DSM 101122	human	O7:H5	ESBL *blaCTX-M1*, *-M15*, *blaTEM*, risk group 2
DSM 101123	human	O7:H5	ESBL, risk group 2
DSM 101124	human urine	O^{NT}:H15	ESBL *blaCTX-M1*, *-M15*, risk group 2
DSM 101125	human	O^{NT}:H9 var. 7	ESBL, risk group 2
DSM 101126	human urine	O75:H9 var. 7	ESBL, risk group 2
DSM 101127	human	O25:H4	ESBL, risk group 2
DSM 101128	human	O25:H4	ESBL, risk group 2
DSM 101129	human urine	O25:H4	ESBL, risk group 2
DSM 101131	human urine	O25:H4	ESBL, risk group 2
DSM 101132	human	O^{rough}:H4	ESBL, risk group 2
DSM 101133	human	O^{NT}:H18	ESBL, risk group 2
DSM 101134	human urine	O7:H5	ESBL, risk group 2
DSM 101135	human urine	O25:H4	ESBL, risk group 2
DSM 101136	human urine	O25:H4	ESBL, risk group 2
DSM 101137	human	O^{NT}:H18	ESBL, risk group 2
DSM 101138	human	O1:H6	ESBL, risk group 2
DSM 101139	human	O1:H6	ESBL *blaCTX-M1*, *-M15*, risk group 2
DSM 101140	human	O^{NT}:H6	ESBL, risk group 2
DSM 101141	human	O15:H1	ESBL, risk group 2
DSM 101142	human	O^{NT}:H4	ESBL, risk group 2

DSM 103242	chicken carcass	O138:H48	ESBL *blaSHV*, risk group 2
DSM 103243	chicken carcass	O25:H48	ESBL *blaSHV*, risk group 2
DSM 103244	chicken carcass	O38:H39	ESBL *blaTEM*, risk group 2
DSM 103245	chicken carcass	O38:H39	ESBL *blaTEM*, risk group 2
DSM 103246	chicken carcass	O186:H34	ESBL *ctxM9*, risk group 2
DSM 103247	chicken carcass	O88:H7	ESBL *blaTEM*, risk group 2
DSM 103248	chicken carcass	O162:H10	ESBL *blaTEM*, risk group 2
DSM 103249	chicken carcass	O^{NT}:H10	ESBL *ctxM9* + *blaTEM*, risk group 2
DSM 103250	chicken carcass	O^{NT}:H25	ESBL *blaCTX-M1*, *blaCTX-M15, risk group 2*
DSM103251	chicken carcass	O91:H7	ESBL *blaCTX-M1*, *blaCTX-M15, risk group 2*
DSM 103252 (ECOR-47)	sheep	O^{NT}:H18	Group D strain
DSM 103253 (ECOR-72)	human, UTI	O144:H8	Group B1 strain
DSM103254	chicken air sac	O78++	APEC; astA -, CDTIII -, CNF1/2 -, FyuA -, irp2 -, hlyA -, aer +, tsh +, fimC +, papC +, hlyE -, stx2f -
DSM103255	chicken peritoneum	O78++	APEC; astA +, CDTIII -, CNF1/2 -, FyuA +, irp2 +, hlyA -, aer +, tsh +, fimC +, papC -, hlyE -, stx2f -
DSM103256	chicken	O2:K1	APEC
DSM103257	chicken wattle	O2:K1	APEC
DSM103258	chicken	O2:K1	APEC
DSM103259	chicken bone marrow	O2	APEC
DSM103260	chicken heart blood	O1:H^{NT}	APEC; astA +, CDTIII -, CNF1/2 -, FyuA +, irp2 +, hlyA -, aer +, tsh +, fimC +, papC +, hlyE -, stx2f -
DSM103261	chicken	O2:K1	APEC
DSM103262	chicken	O1:K1	APEC
DSM103263	chicken heart blood	O78:K80	APEC; aerA+, tsh+
DSM103264	chicken	O1:K1	APEC
DSM103265	dove	O1:H15	APEC; Col-, Hly-,
DSM103266	chicken	O2	APEC; astA-,iss +, irp2 +, papC -, iuc D +, tsh +, vat -, cvi/cva +
DSM103266	chicken	O2	APEC; astA-,iss +, irp2 +, papC -, iuc D +, tsh +, vat -, cvi/cva +

(NM = non motile, NT = non-typeable, ND = no data)

Table S2. *Klebsiella* strains and growth conditions used for host spectrum analyses of phage KWBSE43-6 and Goslar

strain	species	medium	temperature [°C]
DSM 16963	*Klebsiella oxytoca*	1.5% Peptone from casein<br>0.5% Peptone from soymeal<br>0.5% NaCl<br>(0.3/1.5% Agar)	30
DSM 103516-103525	*Klebsiella pneumoniae*	0.5% Peptone<br>0.3% Meat extract<br>(0.3/1.5% Agar)	30
DSM 103700, DSM 103696	*Klebsiella pneumoniae subsp. pneumoniae*		
DSM 102039, DSM 102040	*Klebsiella pneumoniae*		28
DSM 25736, DSM 5175	*Klebsiella oxytoca*		
DSM 29417	*Klebsiella pneumoniae*	3% Trypticase Soy Broth<br>(0.3/1.5% Agar)	37
DSM 24121	*Klebsiella oxytoca*		

Table S3. Additional information on phages isolated in this study

phage	*E. coli* strain used for isolation/propagation (DSM)	origin	geographical origin	date of isolation
Goslar	103255	duck feces	Goslar	May 16
D5505	101114	surface water	Braunschweig	Nov 15
EdH4	498	duck feces	Braunschweig	Feb 16
G10400	103266	pig manure	Hameln	Aug 16
G17	103244	pig manure	Hameln	Aug 16
G2133	103254	pig manure	Hameln	Aug 16
G2248	103255	pig manure	Hameln	Aug 16
G2285	103256	pig manure	Hameln	Aug 16
G2469	103258	pig manure	Hameln	Aug 16
G2494	103259	pig manure	Hameln	Aug 16
G2540	103260	pig manure	Hameln	Aug 16
G2540-3	103260	pig manure	Hameln	Aug 16
G29	103247	pig manure	Hameln	Aug 16
G37-3	103248	pig manure	Hameln	Aug 16
G4498	103261	pig manure	Hameln	Aug 16

G4500	103262	pig manure	Hameln	Aug 16
G4507	103263	pig manure	Hameln	Aug 16
G50	103250	pig manure	Hameln	Aug 16
G5211	103264	pig manure	Hameln	Aug 16
G53	103251	pig manure	Hameln	Aug 16
G8	103243	pig manure	Hameln	Aug 16
G9062	103265	pig manure	Hameln	Aug 16
HdK5	498	chicken feces	Seggebruch	Feb 16
KAW1E185	103253	clinical wastewater	Braunschweig	Apr 16
KAW3E185	103253	clinical wastewater	Braunschweig	Apr 16
KWBSE43-6	103249	sewage	Braunschweig	Jun 17
MM02	498	duck feces	Braunschweig	Aug 14
OE5505	101114	surface water	Braunschweig	Nov 15
R5505	101114	surface water	Braunschweig	Nov 15
Schickermooser	103260	chicken feces	Arnsberg	May 16
WFbE185	103253	sewage	Wolfenbüttel	Feb 16
WFC	101121	sewage	Wolfenbüttel	Dec 15
WFH	101139	sewage	Wolfenbüttel	Dec 15
WFK	101124	sewage	Wolfenbüttel	Nov 15
WFL6982	101124	sewage	Wolfenbüttel	Nov 15
KAW1A4500	103262	clinical wastewater	Braunschweig	Apr 16
PTXU04	613	sewage	Wolfenbüttel	Jan 14
R4596	101113	surface water	Braunschweig	Nov 15
WFI101126	101126	sewage	Wolfenbüttel	Nov 15
EASG3	498	duck feces	Goslar	Feb 16
G29-2	103247	pig manure	Hameln	Aug 16
HASG4	498	chicken feces	Goslar	Feb 16
HdH2	498	chicken feces	Braunschweig	Feb 16
HDK1	498	chicken feces	Seggebruch	Feb 16
HdSG1	498	chicken feces	Goslar	Feb 16
MM01	498	horse feces	Braunschweig	Aug 14
PTXU06	498	sewage	Braunschweig	Jan 14
VAH1	498	budgie droppings	Braunschweig	Feb 16
WFI	103252	sewage	Wolfenbüttel	Feb 16
WF5505	101114	sewage	Wolfenbüttel	Nov 15

viruses

MDPI

Table S4. Morphological and genomic features of isolated phages of this study

Phage	Morphotype	*ICTV genus*	genome size (bp)	CDS	tRNAs	GC content (%)	coding (%)	length TR (bp)	coverage	GenBank acc. no.
vB_EcoM_Goslar	Myovirus	proposed *Goslarvirus*	237,307	247	-	46.53	93.2	-	99.1	MK327938
vB_EcoM_D5505	Myovirus	*Tequatrovirus*	168,409	271	11	35.39	93.8	-	40.6	MK327929
vB_EcoM_EdH4	Myovirus	*Vequintavirus*	136,031	214	7	43.65	90.7	458	700	MK327930
vB_EcoM_G10400	Myovirus	*Tequatrovirus*	170,182	278	7	35.29	95.0	-	300.9	MK327937
vB_EcoM_G17	Myovirus	*Asteriusvirus*	370,817	659	7	34.33	91.1	20,649	203.6	MK327931
vB_EcoM_G2133	Myovirus	*Tequatrovirus*	168,959	275	8	35.33	95.2	-	63.2	MK327928
vB_EcoM_G2248	Myovirus	*Krischvirus*	170,678	277	-	40.52	94.9	-	186.7	MK327932
vB_EcoM_G2285	Myovirus	*Mosigvirus*	166,675	267	2	37.54	94.7	-	295.5	MK327933
vB_EcoM_G2469	Myovirus	*Mosigvirus*	170,452	273	2	37.57	94.2	-	429.3	MK327934
vB_EcoM_G2494	Myovirus	*Krischvirus*	168,327	281	-	40.32	94.1	-	51.6	MK327935
vB_EcoM_G2540	Myovirus	*Tequatrovirus*	168,886	273	8	35.36	94.8	-	536.6	MK327936
vB_EcoM_G2540-3	Myovirus	*Tequatrovirus*	168,654	276	8	35.33	94.8	-	409.9	MK327944
vB_EcoM_G29	Myovirus	*Tequatrovirus*	168,241	276	12	35.33	94.5	-	91.7	MK327940
vB_EcoM_G37-3	Myovirus	*Krischvirus*	167,832	283	-	40.33	93.9	-	456.2	MK327941
vB_EcoM_G4498	Myovirus	*Tequatrovirus*	167,826	280	10	35.46	95.2	-	186.6	MK327939
vB_EcoM_G4500	Myovirus	*Tequatrovirus*	168,363	279	12	35.32	94.7	-	497.8	MK327945
vB_EcoM_G4507	Myovirus	*Tequatrovirus*	168,828	274	12	35.37	94.6	-	381.7	MK327946
vB_EcoM_G50	Myovirus	*Tequatrovirus*	167,728	267	11	35.52	94.5	-	409.9	MK327942
vB_EcoM_G5211	Myovirus	*Krischvirus*	164,278	270	-	40.44	94.0	-	478.2	MK327947
vB_EcoM_G53	Myovirus	*Mosigvirus*	167,834	273	2	37.75	95.0	-	258.6	MK327943
vB_EcoM_G8	Myovirus	*Tequatrovirus*	169,65	268	8	35.33	94.4	-	606.5	MK373787
vB_EcoM_G9062	Myovirus	*Tequatrovirus*	168,67	276	11	35.3	94.8	-	38.7	MK373779
vB_EcoM_HdK5	Myovirus	*Vequintavirus*	139,328	225	7	43.63	90.5	458	424.3	MK373780
vB_EcoM_KAW1E185	Myovirus	*Tequatrovirus*	164,987	274	10	35.37	95.0	-	506.3	MK373781

vB_EcoM_KAW3E185	Myovirus	*Mosigvirus*	170,187	275	2	37.55	95.0	-	60.7	MK373782
vB_EcoM_KWBSE43-6	Myovirus	proposed *Taipeivirus*	158,607	222	7	46.09	92.9	-	51.2	MK373783
vB_EcoM_MM02	Myovirus	*Mosigvirus*	169,201	274	2	37.6	95.0	-	272.9	MK373784
vB_EcoM_OE5505	Myovirus	*Tequatrovirus*	168,756	282	9	35.22	95	-	74.1	MK373785
vB_EcoM_R5505	Myovirus	*Tequatrovirus*	167,317	279	11	35.36	95.2	-	133.1	MK373786
vB_EcoM_Schickermooser	Myovirus	proposed *Phapecoctavirus*	151,194	284	10	39.01	91.3	328	346.2	MK373788
vB_EcoM_WFbE185	Myovirus	*Mosigvirus*	170,429	277	2	37.6	95.1	-	189.9	MK373778
vB_EcoM_WFC	Myovirus	proposed *Wifcevirus*	72,472	113	-	46.01	90.6	3,783	319.8	MK373777
vB_EcoM_WFH	Myovirus	proposed *Wifcevirus*	71,283	106	-	46.12	90.2	3,7	416.5	MK373776
vB_EcoM_WFK	Myovirus	*Mosigvirus*	164,59	262	3	37.58	94.7	-	455.1	MK373775
vB_EcoM_WFL6982	Myovirus	*Mosigvirus*	164,279	261	3	37.56	94.8	-	173.9	MK373774
vB_EcoP_KAW1A4500	Podovirus	*Zindervirus*	44,241	58	-	45.18	90.5	266	774.2	MK373773
vB_EcoP_PTXU04	Podovirus	proposed *Xuquatrovirus*	61,6	92	-	52.84	96.4	-	175.4	MK373772
vB_EcoP_R4596	Podovirus	*Zindervirus*	45,13	51	-	44.84	91.7	428	953.3	MK373771
vB_EcoP_WFI101126	Podovirus	*Kuravirus*	77,307	135	1	42.07	90.2	-	97.9	MK373770
vB_EcoS_EASG3	Siphovirus	*Tequintavirus*	120,715	179	23	39.04	84.8	10,226	591.4	MK373799
vB_EcoS_G29-2	Siphovirus	*Tunavirus*	51,739	85	-	44.02	90.3	-	625.3	MK373798
vB_EcoS_HASG4	Siphovirus	*Tequintavirus*	120,603	178	23	39.02	84.3	10,265	599.4	MK373797
vB_EcoS_HdH2	Siphovirus	*Tequintavirus*	120,12	178	24	39.32	85.4	10,165	393.6	MK373796
vB_EcoS_HDK1	Siphovirus	*Nonagvirus*	64,059	100	-	44.07	89.8	3,48	228.7	MK373794
vB_EcoS_HdSG1	Siphovirus	*Nonagvirus*	63,595	93	-	43.87	91.1	3,362	88.1	MK373795
vB_EcoS_MM01	Siphovirus	*Rtpvirus*	43,157	68	1	43.77	92.2	-	173.5	MK373793
vB_EcoS_PTXU06	Siphovirus	*Dhillonvirus*	42,321	63	-	54.44	92.1	-	1819.6	MK373789
vB_EcoS_VAH1	Siphovirus	*Dhillonvirus*	124,537	208	21	38.61	86.7	11,708	850.5	MK373792
vB_EcoS_WFI	Siphovirus	*Dhillonvirus*	45,536	61	-	54.57	92.5	-	660.4	MK373791
vB_EcoS_WF5505	Siphovirus	*Dhillonvirus*	47,082	67	-	54.3	93.0	2,025	442.9	MK373790

viruses

MDPI

Table S6. Mascot research results for peptide fingerprinting analysis of structural proteins of phages PTXU04 and Goslar

**#analysed band**	**ID**	**Description**	**Score**	**Expect**	**Coverage (%)**	**#Peptides**	**Mass (Da)**
1	PTXU04_00007	hypothetical protein	66	0.00015	7	6	89168
2	PTXU04_00010	putative major capsid protein	192	3.7e-017	52	14	36442
3	PTXU04_00010	putative major capsid protein	70	5.4e-005	26	8	36442
	PTXU04_00024	hypothetical protein	171	4.7e-015	56	16	30776
4	PTXU04_00012	hypothetical protein	58	0.001	63	3	13847
5	PTXU04_00011	hypothetical protein	74	2.6e-005	71	5	7076
1	Goslar_00217	hypothetical protein	215	1.92-019	25	19	76826
2	Goslar_00041	hypothetical protein	219	7.4e-020	28	25	83540
3	Goslar_00247	hypothetical protein	81	5.1e-006	31	11	50985
4	Goslar_00060	hypothetical protein	49	0.0074	26	7	34049
5	Goslar_00218	hypothetical protein	82	4.6e-006	52	9	33055
6	Goslar_00022	hypothetical protein	143	3e-012	65	14	32458
7	Goslar_00058	hypothetical protein	114	2.4e-009	28	10	47103
8	Goslar_00061	hypothetical protein	54	0.0023	15	6	40210
	Goslar_00247	hypothetical protein	42	0.035	17	5	31408
9	Goslar_00023	hypothetical protein	81	4.4e-006	37	5	19113

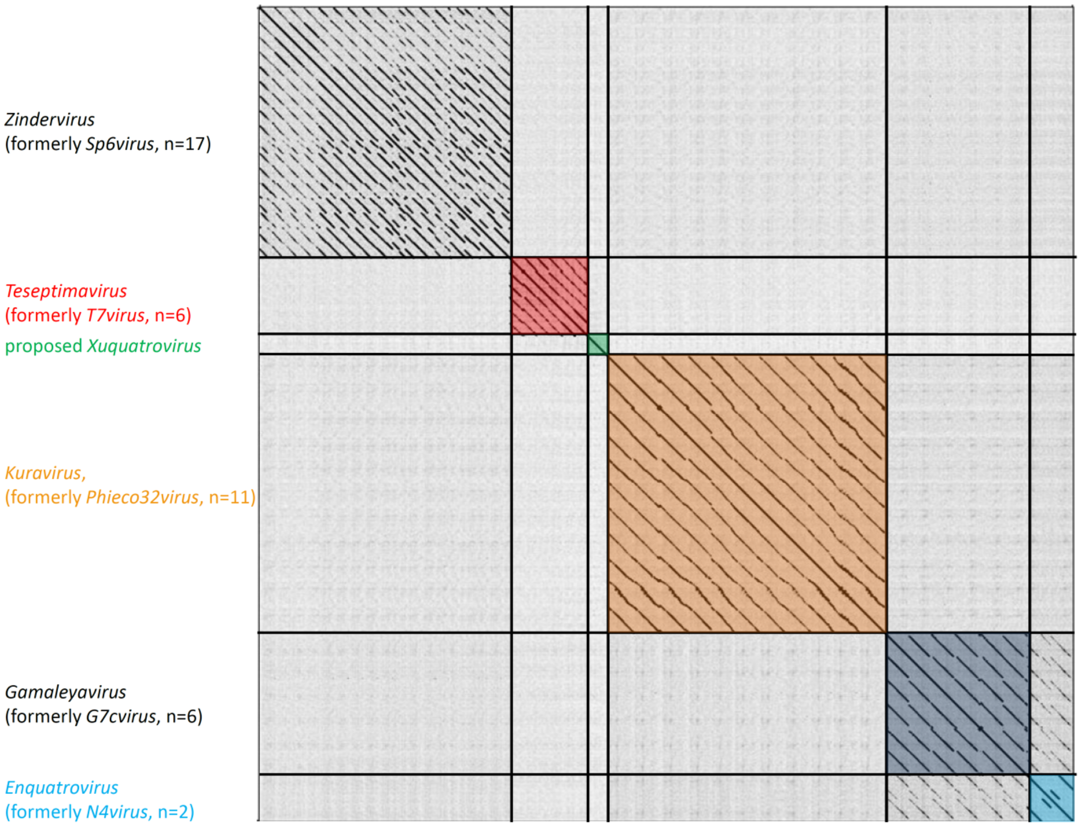

Suppl. Figure S1 Dot plot analysis of genomes of isolated podoviruses of this study compared to related genomes and further members of different genera of the *Podoviridae* family. Genomes were colinearized before analysis. Figure was generated using GEPARD.

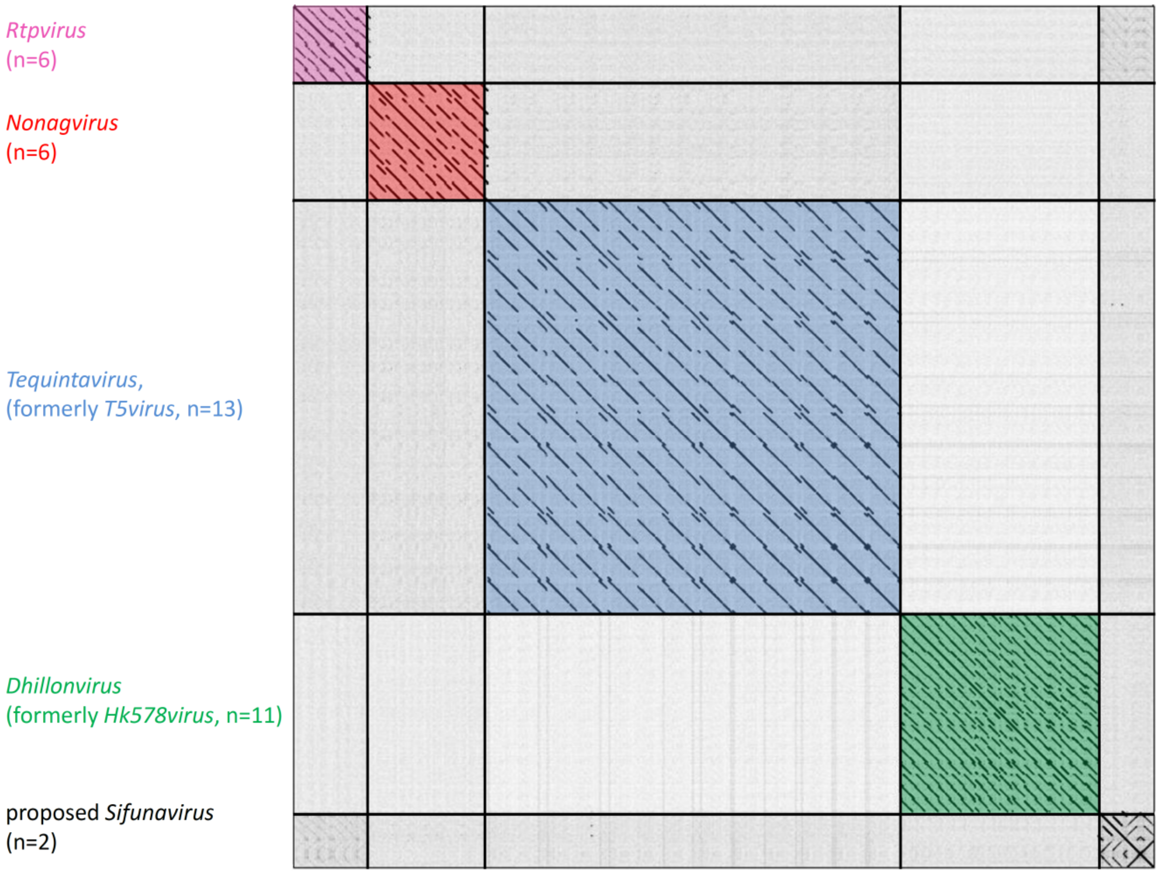

Suppl. Figure S2 Dot plot analysis of genomes of isolated siphoviruses of this study compared torelated genomes and further members of different genera of the *Siphoviridae* family. Genomes were colinearized before analysis. Figure was generated using GEPARD.

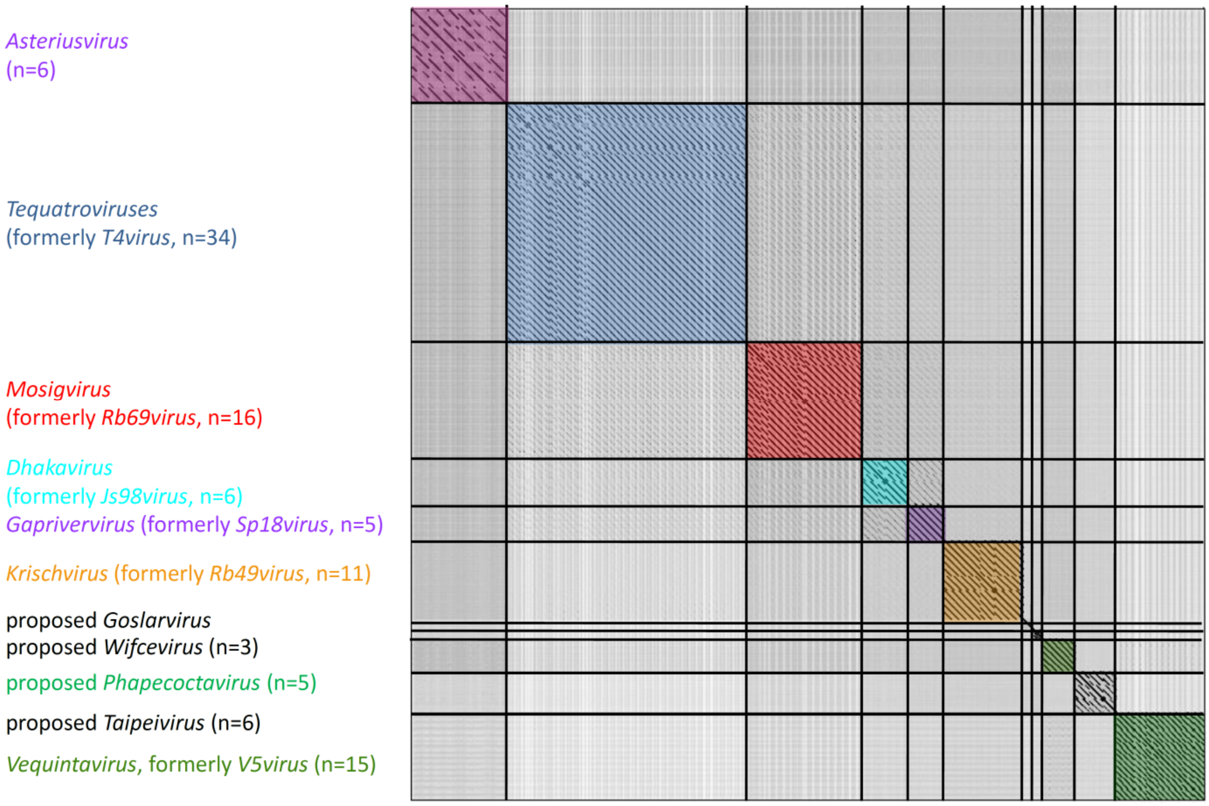

Suppl. Figure S3 Dot plot analysis of genomes of isolated myoviruses of this study compared to related genomes. Genomes were colinearized before analysis. Figure was generated using GEPARD.

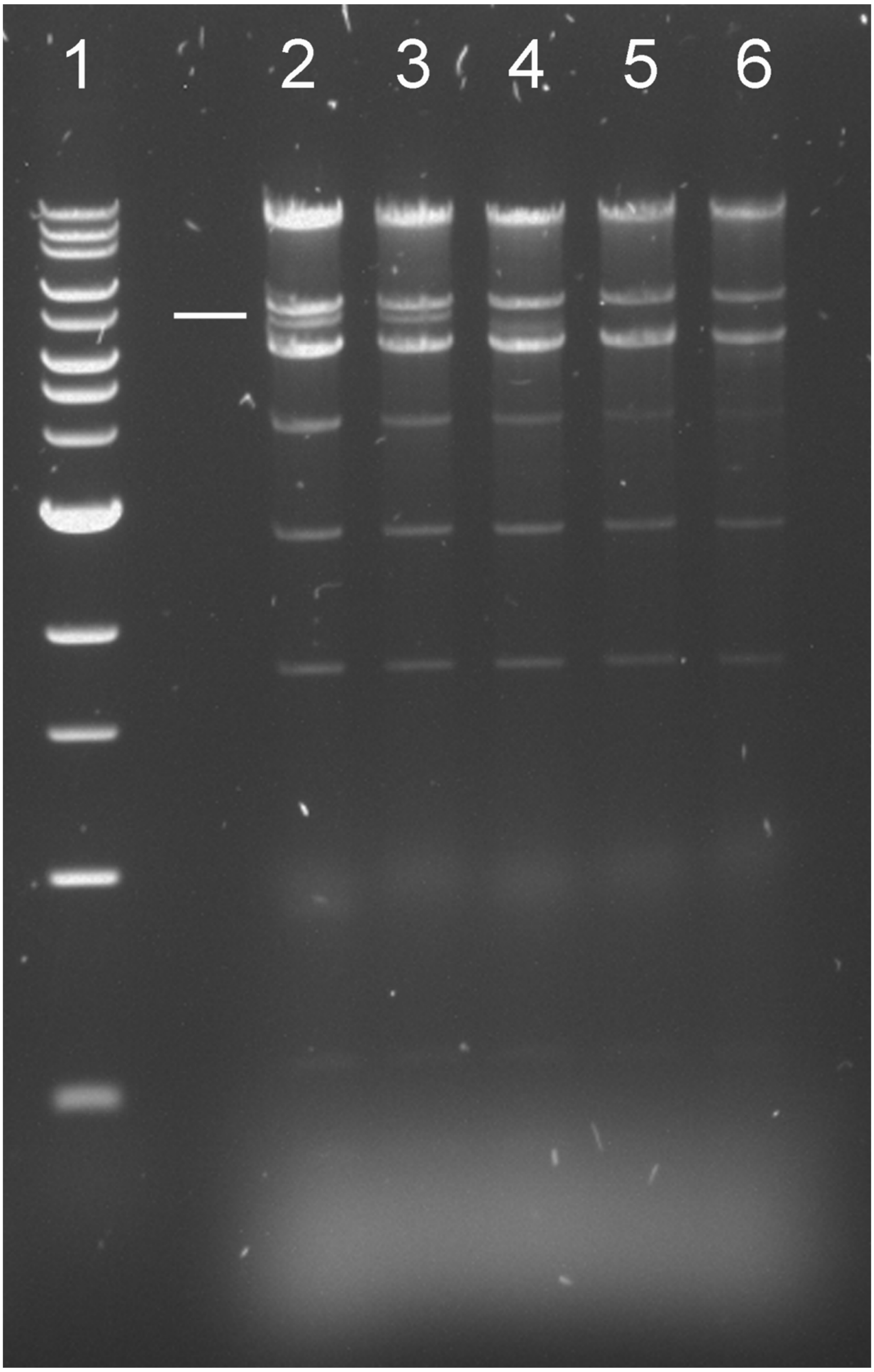

Suppl. Figure S4 Time-limited digestion of genomic DNA of phage PTXU04 with BAL 31, followed by complete hydrolysis with BglI. Lane 1, Marker (Quick load 1kb Extended, Bio labs); lane 2–6, DNA of PTXU04 hydrolyzed with BAL 31 followed by BglI. BAL 31 digestion time: lane 2, 0 min; lane 3, 15 min; lane 4, 30 min; lane 5, 45 min. lane 6, 60 min. 1% agarose gel. Band representing predicted and degraded end is marked.

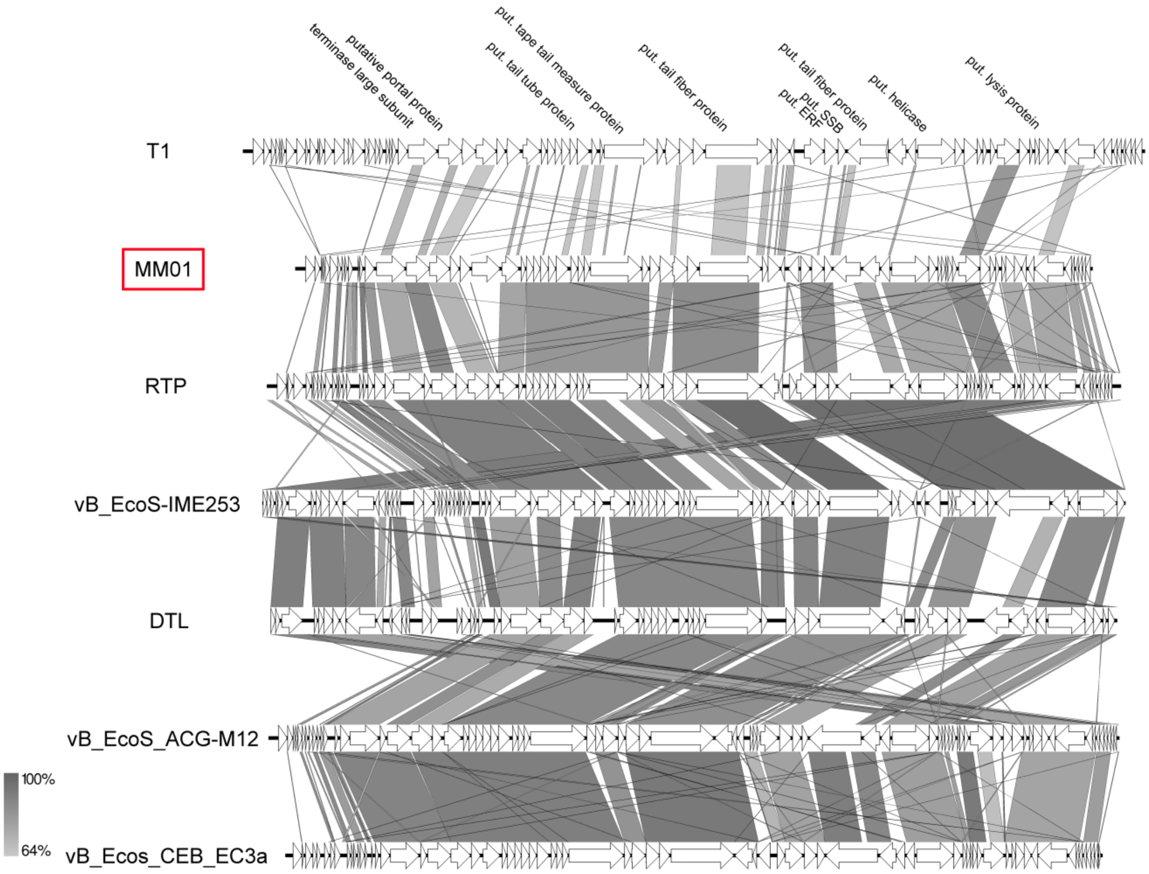

Suppl. Figure S5. Synteny plot of phage MM01 compared to related phages of the rtpviruses and phage T1 at the nucleotide level. Figure was generated with EasyFig, isolate of this study is marked in red.

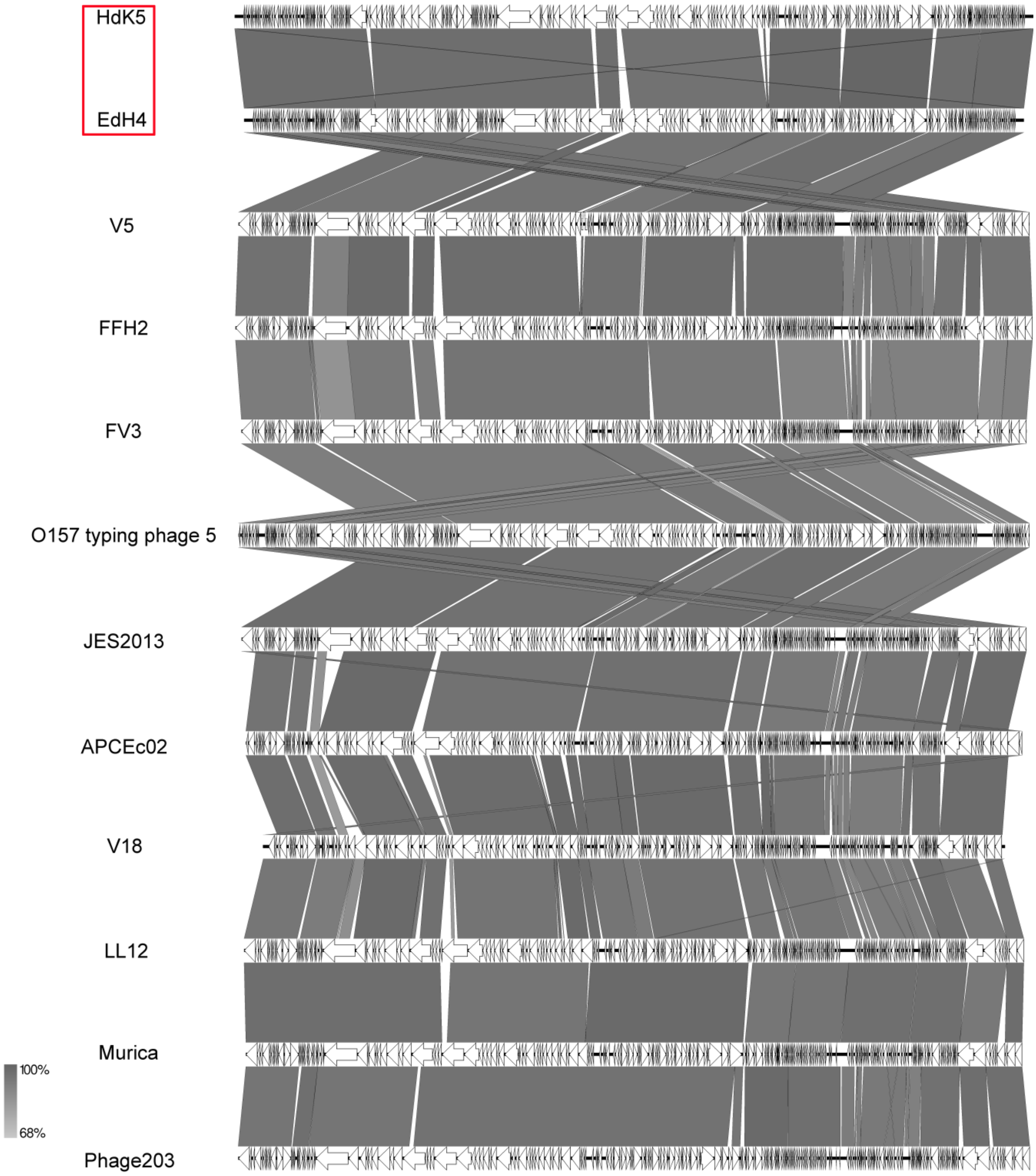

Suppl. Fig S6. Genomic organization of phages EdH4 and Hdk5 compared to related viruses at the nucleotide level. Figure was generated with EasyFig, isolate of this study is marked in red.

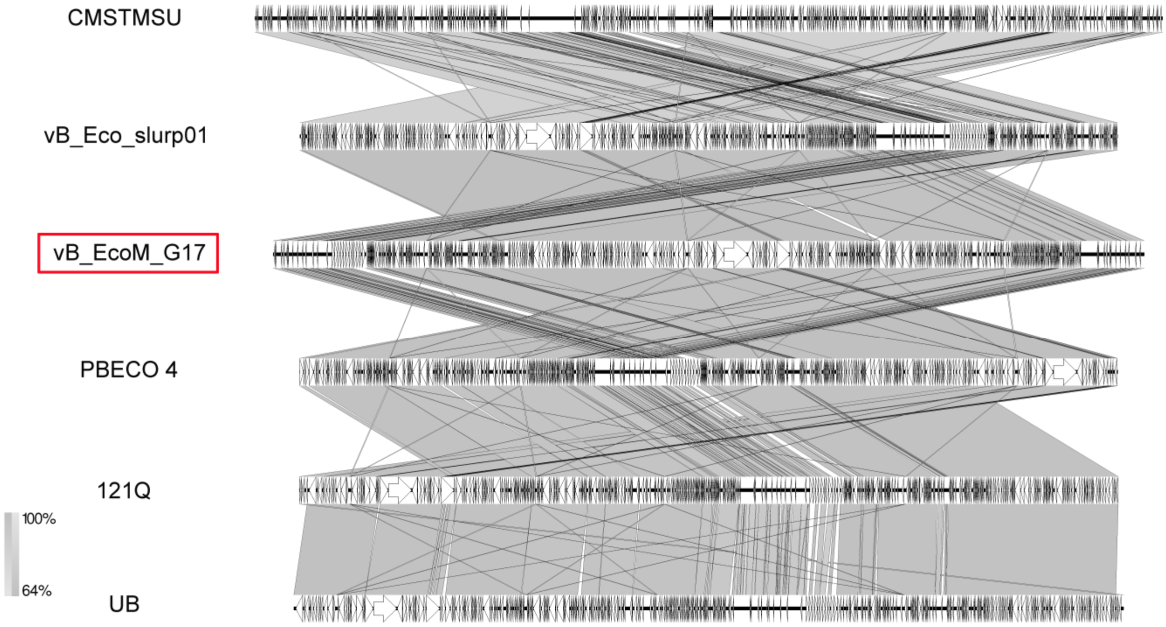

Suppl. Fig S7. Synteny plot of phage G17 compared to related asteriusviruses at the nucleotide level. Figure was generated with EasyFig, isolate of this study is marked in red.

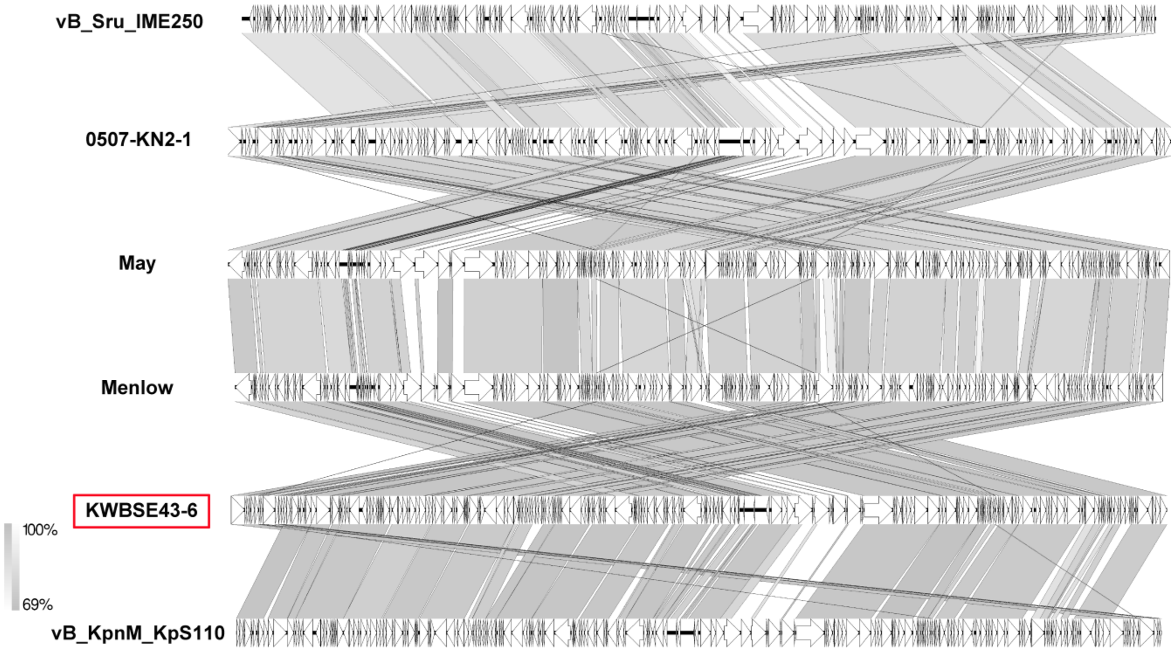

Suppl. Fig S8. Genome organization of phage KWBSE43-6 compared to related phages at the nucleotide level. Figure was generated with EasyFig, isolate of this study is marked in red.

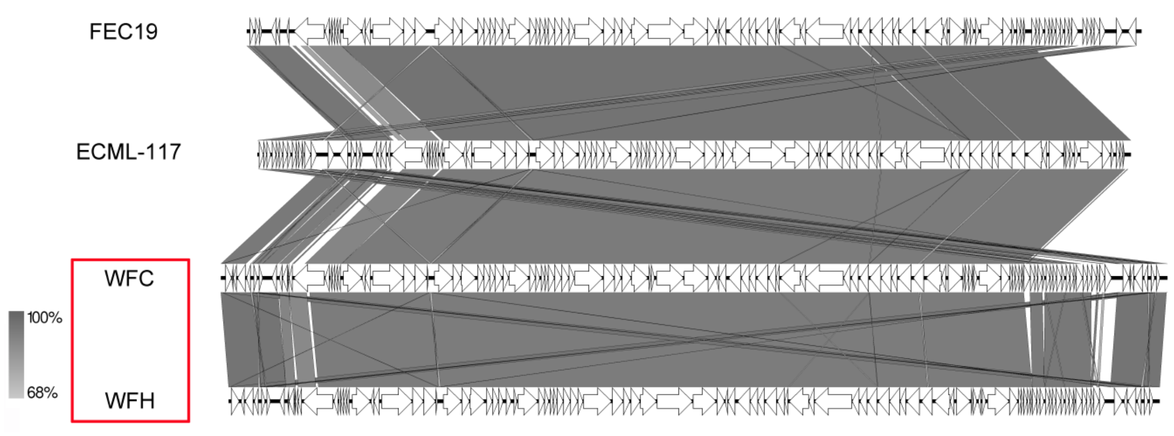

Suppl. Fig S9. Synteny plot of phage WFC and WFH compared to related phages at the nucleotide level. Figure was generated with EasyFig, isolates of this study are marked in red.

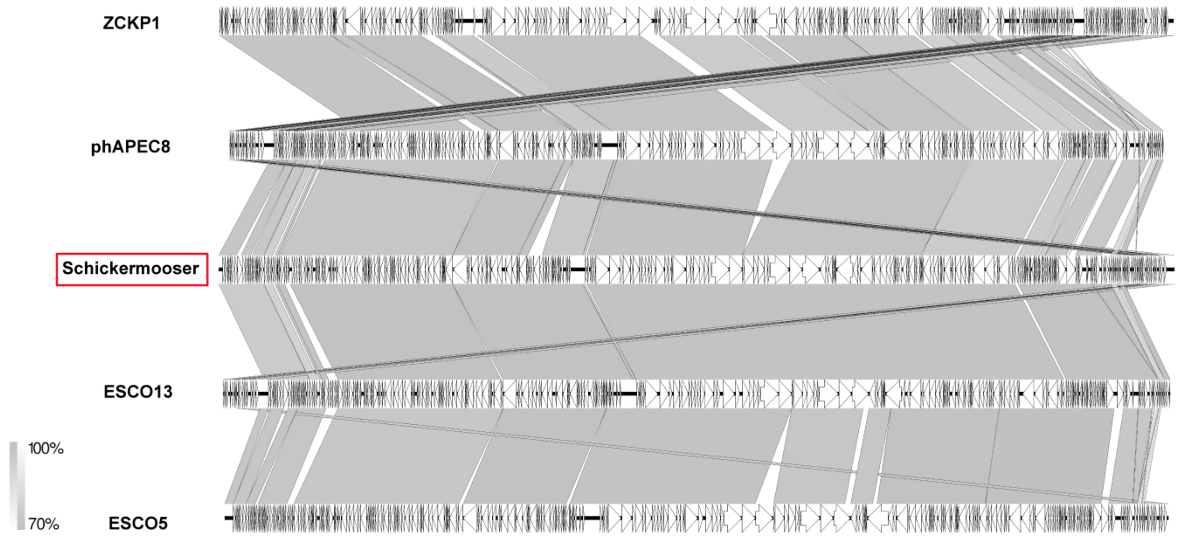

Suppl. Fig S10. Synteny plot of phage Schickermooser compared to related phages at the nucleotide level. Figure was generated with EasyFig, isolate of this study is marked in red.
